# Multiplexed neuropeptide mapping in ant brains integrating microtomography and three-dimensional mass spectrometry imaging

**DOI:** 10.1093/pnasnexus/pgad144

**Published:** 2023-04-25

**Authors:** Benedikt Geier, Esther Gil-Mansilla, Zita Liutkevičiūtė, Roland Hellinger, Jozef Vanden Broeck, Janina Oetjen, Manuel Liebeke, Christian W Gruber

**Affiliations:** Department of Symbiosis, Max Planck Institute for Marine Microbiology, Bremen 28359, Germany; Department of Pediatrics and Infectious Diseases, Stanford School of Medicine, Stanford, CA 94305, USA; Center for Physiology and Pharmacology, Medical University of Vienna, Vienna 1090, Austria; Center for Physiology and Pharmacology, Medical University of Vienna, Vienna 1090, Austria; Center for Physiology and Pharmacology, Medical University of Vienna, Vienna 1090, Austria; Molecular Developmental Physiology and Signal Transduction Group, Zoological Institute, KU Leuven, Leuven 3000, Belgium; Bruker Daltonics GmbH & Co. KG, Life Science Mass Spectrometry, Bremen 28359, Germany; MALDI Imaging Lab, University of Bremen, Bremen 28359, Germany; Department of Symbiosis, Max Planck Institute for Marine Microbiology, Bremen 28359, Germany; Department of Metabolomics, Institute of Human Nutrition and Food Science, Kiel University, 24118 Kiel, Germany; Center for Physiology and Pharmacology, Medical University of Vienna, Vienna 1090, Austria

## Abstract

Neuropeptides are important regulators of animal physiology and behavior. Hitherto the gold standard for the localization of neuropeptides have been immunohistochemical methods that require the synthesis of antibody panels, while another limiting factor has been the brain's opacity for subsequent in situ light or fluorescence microscopy. To address these limitations, we explored the integration of high-resolution mass spectrometry imaging (MSI) with microtomography for a multiplexed mapping of neuropeptides in two evolutionary distant ant species, *Atta sexdens* and *Lasius niger*. For analyzing the spatial distribution of chemically diverse peptide molecules across the brain in each species, the acquisition of serial mass spectrometry images was essential. As a result, we have comparatively mapped the three-dimensional (3D) distributions of eight conserved neuropeptides throughout the brain microanatomy. We demonstrate that integrating the 3D MSI data into high-resolution anatomy models can be critical for studying organs with high plasticity such as brains of social insects. Several peptides, like the tachykinin-related peptides (TK) 1 and 4, were widely distributed in many brain areas of both ant species, whereas others, for instance myosuppressin, were restricted to specific regions only. Also, we detected differences at the species level; many peptides were identified in the optic lobe of *L. niger*, but only one peptide (ITG-like) was found in this region in *A. sexdens*. Building upon MS imaging studies on neuropeptides in invertebrate model systems, our approach leverages correlative MSI and computed microtomography for investigating fundamental neurobiological processes by visualizing the unbiased 3D neurochemistry in its complex anatomic environment.

**Significance Statement**

Mass spectrometry imaging (MSI) has enabled label-free mapping of molecules in situ and without prior knowledge. Consequently, MSI holds incredible potential for elucidating the function of small molecules, such as neuropeptides, in environmental samples including insects and other invertebrates. However, few studies have integrated the chemical maps with the anatomically and physiologically relevant areas, critical for interpreting the complex neurochemical processes. We designed an integrated high-resolution three-dimensional (3D) MSI and microtomography workflow to advance the field of correlative MSI and enable researchers to integrate neuropeptide distributions with the detailed 3D anatomy when applying MSI. We used two ant species with drastically different morphology and lifestyles as social insect models to reconstruct how neuropeptides distribute within their unmodified 3D neuroanatomy.

## Introduction

Neuropeptides are small molecules that act as extracellular signals regulating important biological processes, such as metabolism, development, reproduction, behavior, and learning. The origin of neuropeptides is deeply rooted in metazoan evolution and likely emerged at very early stages of nervous system development (1).

In the animal kingdom, insects appeared ∼479 million years ago (2) and represent the most diverse group in terms of numbers of described species. This taxon has an enormous significance with respect to ecology, biology, and economy. In fact, insects participate in ecosystem maintenance, act as vectors of diseases, parasitize other animals and plants, pollinate our fruits, or eat our crops with drastic impacts on agriculture. Among insects, social insects such as ants play a vital role in ecosystem functioning worldwide. Ants in particular display a distinguished social colony organization between species and unique caste systems within species, making them an emerging model system that is highly suitable for studying neuropeptide signaling (3). For instance, individuals from the same species belonging to differently sized colonies can exhibit a different brain anatomy depending on the tasks they develop (4, 5). To understand the molecular processes underlying the social behavior in different developmental stages, castes, and species, a link has to be established between neuropeptide chemistry and brain anatomy at the level of individual organisms.

Recently, mining of published genome and transcriptome sequences of ants and other insects has resulted in the prediction of hitherto undescribed neuropeptides (6, 7). Their role, tissue expression, and distribution remain unknown, except for a few, evolutionarily, and behaviorally distinct model species (6, 8). Elucidating the biological function of neuropeptides could provide targets for insect pest management (9) and drug development (10, 11).

Detection and localization of neuropeptides are classically achieved by immunohistochemistry (IHC) and in situ hybridization (12–15). However, these methods require the production of specific labels such as antibodies or complementary genetic probes, respectively. Particularly for multilabeling approaches (e.g. for detection of a set of neuropeptides), design and testing of the individual labels are highly time-consuming and conventionally limited by the number of fluorophores within a single labeling experiment.

Mass spectrometry imaging (MSI) in contrast is a multiplexed and label-free technique, which can map the distribution and relative abundance of hundreds to thousands of molecules from the same tissue section (16). Over the last decade, matrix-assisted laser desorption/ionization (MALDI)-MSI has revolutionized spatial biology (17). The field of neurochemistry has tremendously profited from the ability to detect a variety of molecular species, such as lipids and neurotransmitters, within a single measurement (18). With the ability to measure neuropeptides, scientists have begun to image neuropeptides from diverse animals (for overview, see Table S1). This includes neuropeptide imaging in mammals like rats, mice, primates, and humans and also invertebrates like mollusks, crustaceans, and insects.

One major challenge using MSI on submillimeter-sized tissue samples like insect brains is to properly orient and connect the generated neuropeptide maps to the underlying histology of tissues and cells. Most studies show optical images of the tissue section used for neuropeptides mapping. Only few studies have supplemented their MSI data with detailed histology as well as orientation of the tissue section in 3D space. Instead of complementary histology, Pratavieira et al. (19) projected the MALDI-MSI images onto a schematic that allowed them to assign the detected neurotransmitters to specific regions of the honey bee brain. Others compared IHC with MSI to study the cellular localization of neuromodulators during the development in locusts (20) and in a multiomic approach revealed the peptide chemistry in brain of the ant *Cataglyphis nodus* (21). MSI of serial sections allowed studying the distribution of neuropeptides across the whole brain of a Jonah crab (22).

Noninvasive three-dimensional (3D) imaging techniques such as magnetic resonance tomography (MRT) in combination with MSI provided an improved integration of molecular and anatomic visualization data (23–25). 3D models have been used as virtual atlases, providing an anatomic framework for the coregistration with data sets of other imaging techniques, such as MS and fluorescence imaging data (26). For resolving the neuroanatomy of millimeter-sized arthropods, MRT does not provide the required spatial resolution. Therefore, microscopy-based imaging, such as confocal and/or laser scanning microscopy, has been widely used for insect neuroimaging (27). However, the penetration depth of the microscopy laser, for instance, is limited to 100–300 *µ*m in clear tissue samples and even less in opaque specimens, which means that penetrating the different types of head capsules that encase the arthropods’ brains, if not dissected, poses a substantial challenge.

Microcomputed tomography (µCT) is a X-ray-based, noninvasive 3D imaging technique capable of penetrating through any biological specimen, and unlike most microscopy-based methods, µCT provides the same spatial resolution along *x*-, *y*-, and *z*-axes (28, 29). In insects, other arthropods, and even fossils, high-resolution 3D µCT imaging has been used to resolve taxonomic relations and to study the functional morphology (30–33). Although µCT is X-ray based and not limited in penetration depth, X-rays are poorly attenuated through animal soft tissues. Similar to electron microscopy, tissues are conventionally contrasted with heavy element agents (34). The development of more elaborate contrasting protocols (35, 36) and advances in spatial resolution <1 *µ*m have promoted applications of µCT beyond small-animal imaging, for instance with microscale 3D neuroanatomy reconstruction for the honey bee (28, 37). Furthermore, faster tomographic reconstructions now allow for effective screenings of small animal models supporting physiological and developmental studies (29, 38–41).

Given the spatial resolution advances in both MALDI-MSI and µCT, we reasoned that combining both techniques could provide a powerful link for complementing neuropeptide chemistry with brain anatomy to build maps that advance our understanding of neurophysiology across castes and species of ants and potentially many other animals.

In this study, we optimized a spatial peptidomics procedure by combining MALDI-MSI and µCT in two ant species, the leafcutter ant *Atta sexdens* and the black garden ant *Lasius niger*, resulting in high-resolution molecular neuropeptide mapping and anatomical 3D imaging. This correlative approach enabled us to integrate the spatial distribution of neuropeptides from all planes of the ant brain into a 3D anatomy model and link subregions of the brain with their specific neuropeptide chemistry. We advance previous separate MSI and µCT data sets (26, 42) by integrating them in a full 3D approach that provides unprecedented detail of ant brain anatomy in correlation to neuropeptide distribution.

## Results and discussion

### Development of a workflow for neuropeptide imaging in social insects

**Fig. 1.**
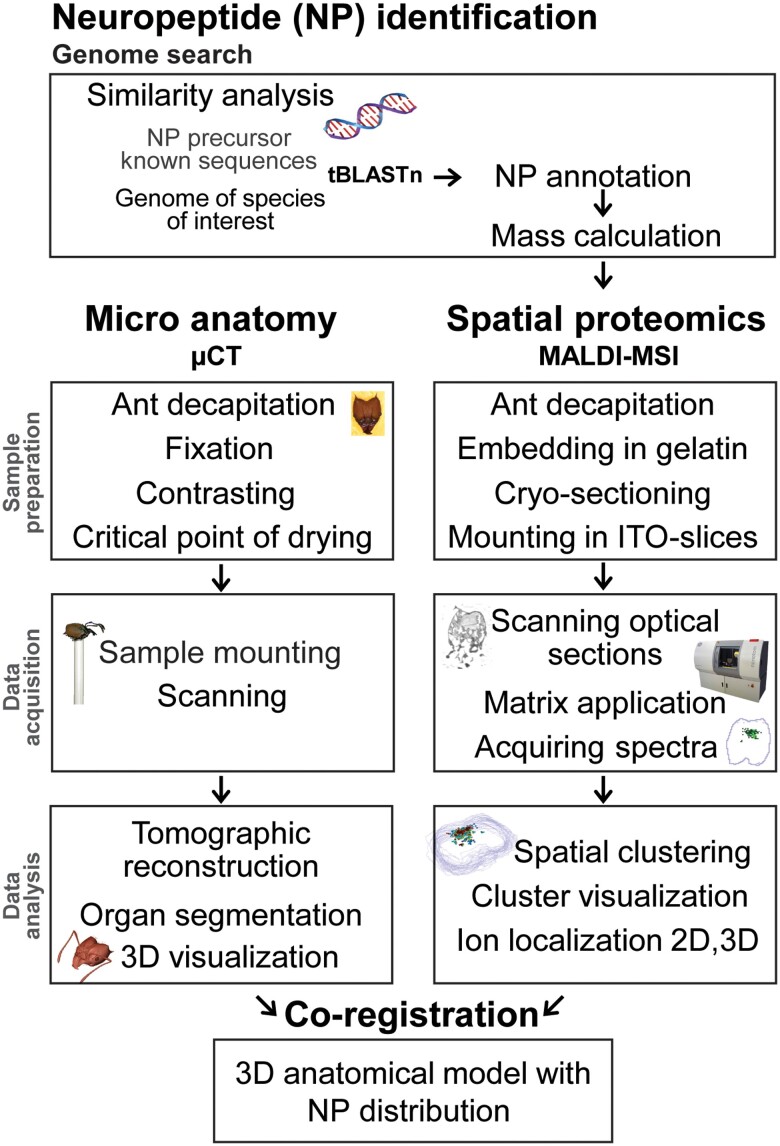
Workflow for genome-based neuropeptide identification and localization with correlative 3D imaging of brain anatomy and spatial peptidomics. Separate sample preparation, imaging, and data analysis pipelines of the µCT and MALDI-MSI data; microanatomy (via µCT) and spatial peptidomics (MALDI-MSI) were acquired from two separate samples of the same species and comparable life stages; coregistration of the µCT and MALDI-MSI data sets in AMIRA was based on manual expert annotation by matching brain areas between virtual µCT slices and optical images of the tissue sections used for MALDI-MSI.

We developed a combined methodological approach to link 3D spatial peptidomics to full brain microanatomy (see Fig. 1). Thereby, we focused on two species from different positions in the ant evolutionary tree (43) which display substantial differences in head morphology and social behavior. The first step of our approach was the identification of neuropeptides by dereplication present in each of the two model ant species, *L. niger* and *A. sexdens*. We mined four available ant genome data sets to predict neuropeptide sequences of the two model species in this study. From the amino acid sequences of each neuropeptide, we calculated the theoretical *m/z* values. These target *m/z* values were subsequently mapped using MALDI-MSI data from on a series of consecutive ant brain sections. In parallel, a comparable head in terms of size and caste of each species was contrasted and imaged by µCT, resulting in a 3D anatomical model. Finally, the spatial peptidomics data were manually coregistered into the anatomic 3D model in a virtual space resulting in a 3D atlas correlating anatomy with neuropeptide biochemistry for direct comparison and localization.

### In silico prediction, sequencing, and in situ detection of ant neuropeptides

For the in silico predictions, we compared published neuropeptide precursor sequences of *Acromyrmex echinatior* (44) and *Camponotus floridanus* (45) with the genome of *L. niger* (46) on one hand and *Atta cephalotes* (47), a species closely related to *A. sexdens*, on the other hand as the genome and transcriptome of *A. sexdens* ant are not available. In the genome of each species, we discovered 30 neuropeptide precursors, which encode 58 neuropeptides in *L. niger* and 57 in *A. cephalotes* (Table S2). Furthermore, we confirmed the allatostatin (AST) A, myosuppressin (MS), tachykinin-related peptide (TK), and short neuropeptide F (sNPF) precursor sequences by molecular cloning to further validate the in silico prediction of neuropeptide data set (Table S2 and supplementary material S1). Particular, *L. niger* neuropeptide sequences (13 of in total 15), which were encoded by these four cloned precursors, matched the ones obtained via in silico prediction. After comparing mature peptide sequences of different ants, we found that myrmicine species *A. echinatior* and *A. cephalotes* share 65% of the peptides with identical sequences, whereas *A. echinatior* and the formicine *L. niger*—evolutionary more distant species (48)—exhibited only 25% sequence identity at neuropeptide level. Since *A. cephalotes* and *A. sexdens* belong to the same genus, we could expect high sequence conservation and neuropeptide similarities. After cloning four precursors and a comparative analysis of the neuropeptide sequences, we confirmed that the amino acid sequences for 10 out of 13 peptides were identical in both species, resulting overall in 76% neuropeptide identity between the two model ant species. We found the biggest difference within the precursor of TKs in *A. sexdens*, which was 129 amino acids shorter compared with *A. cephalotes* and lacked two out of seven TKs. Additionally, the TK7 in *A. sexdens* was not identical to TK7 in the other species but contained one additional serine (Fig. S1). Our approach showed that high-quality prediction of neuropeptides by in silico mining can be complemented through confirmation of peptide-specific masses via MALDI-MSI analysis.

### 3D localization of neuropeptides in situ with serial MALDI-MSI (spatial peptidomics)

Neuropeptides either are conventionally detected in bulk brain extracts, using multiplexed approaches such as liquid chromatography–mass spectrometry (LC–MS/MS) or are visualized individually through specific fluorescent labels using IHC. Using a MSI setup with high-mass resolving power, a magnetic resonance mass spectrometry (MRMS) instrument for untargeted MALDI-MSI enabled us to measure and compare the exact molecular mass of each detected neuropeptide in the tissues. MALDI-MSI enabled us to simultaneously resolve the spatial distribution of up to 15 neuropeptides from measurements of multiple sections derived from the two ant species *L. niger* and *A. sexdens*. Extending MALDI-MSI into a serial approach for spatial neuropeptidomics revealed the need for 3D MSI to cover the spatial diversity of neuropeptides throughout different regions of the brain. The lists of detected molecular masses were used to target the genome-predicted neuropeptide masses (Table S2) of *L. niger* and *A. sexdens* and to annotate and localize each of the neuropeptides (see Table 1).

**Table 1.** Detected neuropeptides in the two analyzed ant species.

	*Lasius niger*				*Atta sexdens*			
Neuropeptide	Peptide sequence^a^	HRM. [M + H]^+^			Peptide sequence	HRM. [M + H]^+^		
		Calc.	Measured	Error (ppm)		Calc.	Measured	Error (ppm)
*Allatostatin A* (*AST A*)								
AST A1	LPLYNFGI^b^	935.535	n.f.		LPLYTFGI^b,a^	922.540	n.f.	
AST A2	TRPFSFGI^b,a^	923.510	923.513	3.2	TRQFSFGI^b,a^	954.516	954.516	0
AST A3	LRDYRFGI^b,a^	1,038.584	1,038.584	0	LRNYDFGI^b,a^	996.526	n.f.	
AST A4	GGKPFSFGI^b^	908.499	n.f.		GNHQFGFGI^b,a^	975.480	n.f.	
AST A5	GWKLATGETAVS^b^	1,218.648	n.f.		VWKLATGETAVS^b,a^	1,260.695	n.f.	
*CAPA*								
CAPA1	SAGLVPYPRI^b^	1,071.631	1,071.634	2.8	SAGLVAYPRI^b^	1,045.615	n.f.	
CAPA2	ALGIIHQPRI^b^	1,116.700	1,116.704	3.6	AFGIIHKPRIG^b^	1,207.742	n.f.	
*Corazonin (CRZ)*								
CRZ	pQTFQYSRGWTN^b^	1,369.629	n.f.		pQTFQYSRGWTN^b^	1,369.629	1,369.631	1.4
*Ecdysis triggering hormone* (*ETH*)								
ETH1	DEVPAFFLKIAKIPTLPRV^b^	2,153.284	2,153.286	0.9	EEVPAFFLKIAKIPTLPRV^b^	2,167.300	n.f.	
IDLSRF-like	IDLSRFYGHINT	1,435.733	1,435.736	2.0	IDLSRFYGHFNT	1,469.717	1,469.720	2.0
ITG-like	ITGQGNRLF	1,005.548	1,005.551	3.0	ITGQGNRLF	1,005.548	1,005.548	0
*Myosuppressin* (*MS*)								
MS	pQDVDHVFLRF^b,a^	1,257.638	1,257.642	3.4	pQDVDHVFLRF^b,a^	1,257.638	1,257.639	0.7
*Neuropeptide-like precursor 1* (*NPLP1*)								
NPLP1 2	NVGTLARDFALPT^b^	1,373.754	1,373.751	2.2	NVGALARDFALPT^b^	1,343.743	n.f.	
NPLP1 3	HIASVARDHGLPN^b^	1,385.740	1,385.746	4.3	HIGSVLRDYSTMS^b^	1,464.726	n.f.	
NPLP1 4	NIGSLARQSTLPSN^b^	1,456.787	1,456.794	4.5	NIGSLARQSMLPIS^b^	1,485.821	1,485.822	0.7
NPLP1 6	NVAALARDSSLPY^b^	1,375.733	1,375.732	0.7	NVAALARDSSLPY^b^	1,375.733	1,375.735	1.5
*Orcokinin* (*OK*)								
OK3	NFDEIDRVGWGGFV	1,610.760	n.f.		NFDEIDRAGWGGFV	1,582.728	1,582.732	2.5
*Pyrokinin* (*PK*)								
PK1	TTAQEITSGMWFGPRL^b^	1,793.900	n.f.		TTQDITSGMWFGPRL^b^	1,708.848	1,708.852	2.3
*Short neuropeptide F* (*sNPF*)								
sNPF	SPSLRLRF^b,a^	974.589	974.592	3.1	SPSLRLRF^b,a^	974.589	974.589	0
*Tachykinin-related peptides* (*TK*)								
TK1	APMGFQGMR^b,a^	993.476	993.480	2.8	APMGFQGMR^b,a^	993.476	993.476	0
TK2	n.f.				APKGFQGMR^b,a^	990.530	990.531	1.0
TK3	LLAMGFQGIR^b,a^	1,104.635	n.f.		ASMGFQGMR^b,a^	983.455	983.456	1.0
TK4	TVMGFQGMR^b,a^	1,025.502	1,025.504	2.0	TLMGFQGMR^b,a^	1,039.518	1,039.518	0
TK7	AAMGFYGTR^b,a^	972.472	972.472	0	AALGFYGTR^b,a^	954.516	954.516	0

^a^Confirmed by cloning; ^b^Amidated C-terminus; calc., calculated; HRM, high-resolution mass; n.f., not found; p, pyroglutyminated N-terminus.

The annotated neuropeptides were encoded by 10 prepropeptide genes in the central nervous system. For *L. niger*, we annotated 16 neuropeptides by MALDI-MSI, which encompass 27.5% of the 58 neuropeptides predicted by genome mining. For eight neuropeptide sequences derived from *L. niger*, we confirmed the expression by precursor cloning and sequencing. For *A. sexdens*, the searches detected 15 *m/z*-values in the MALDI-MSI data, which matched the predicted neuropeptide masses, of which 12 were validated by cloning and sequencing of the precursor (Table 1). A limitation arose due to the same atomic composition of C_44_H_68_N_13_O_11_ for AST A2 and TK7 of *A. sexdens*. Therefore, we could not differentiate these two neuropeptides with MALDI-MSI based on detected high-resolution *m/z* values on the MS1 level in this ant species. LC–MS/MS would have allowed the differentiation of both peptides, but localization would have been impossible after tissue homogenization. MALDI-MSI can be used in MS/MS mode but is limited for low abundant analytes like the neuropeptides. Advances in spatial peptidomics include coupling laser-capture microscopy for micrometer-scale dissection of tissues with LC–MS/MS to maintain spatial coordinates (49). In MSI, ion mobility has become a frontier to separate ions with the same mass based on their mobility in the gas phase prior to MS1 detection (50). Integrating ion-mobility MALDI-MSI with our approach could help to differentiate the distributions of peptides with an identical mass.

In summary, 58 neuropeptides were predicted by genome mining of the related species, of which as a further layer of evidence we cloned and sequenced 12 neuropeptides in *A. sexdens*. From these 12 sequenced neuropeptides in *A. sexdens*, we annotated eight by exact mass determination (see Table 1).

**Fig. 2.**
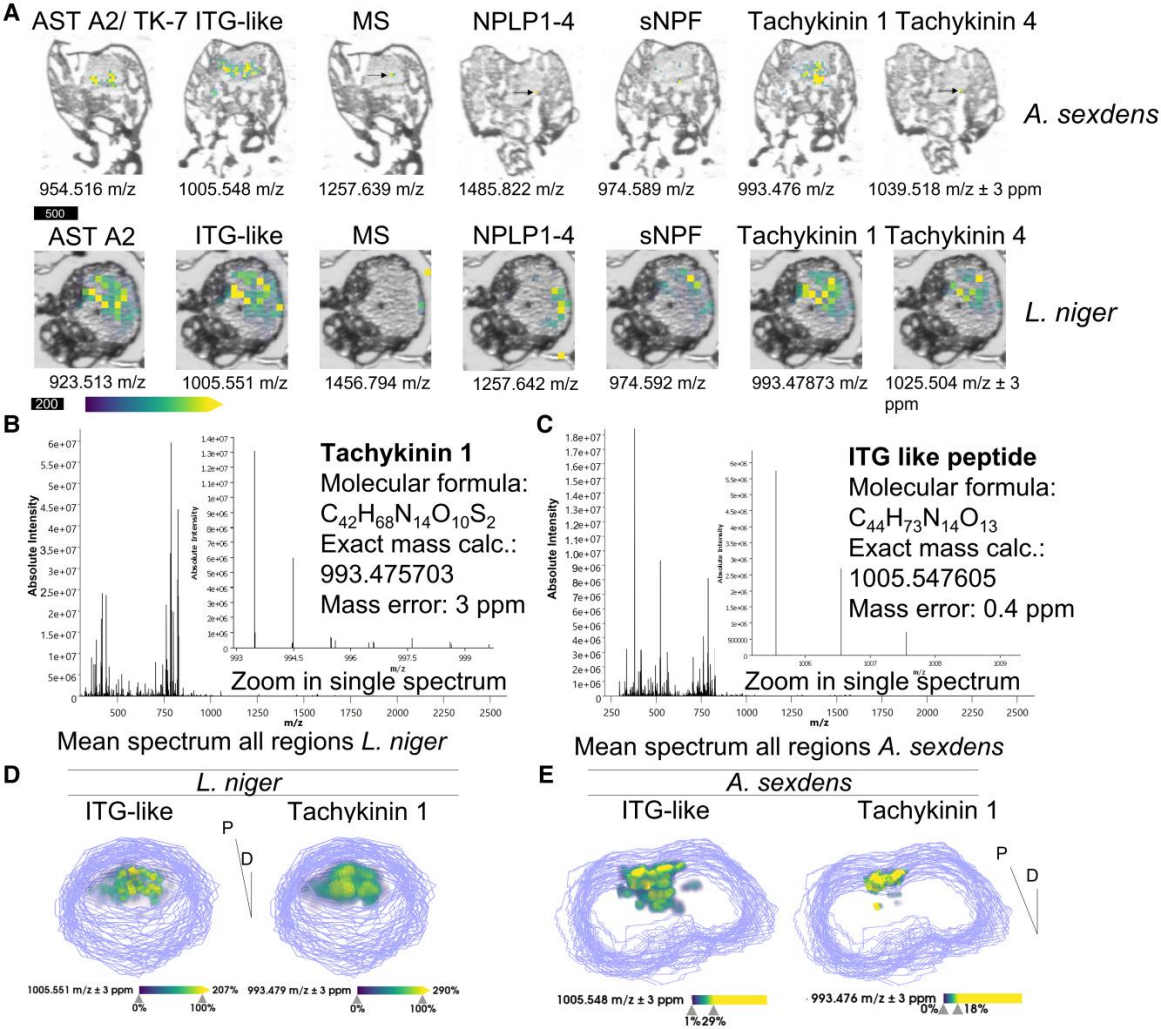
Mass spectrometry imaging maps of neuropeptides in ant brain tissue sections. A) Neuropeptide maps for seven different peptides detected in both, *A. sexdens* (upper panel, scale bar 500 *µ*m) and *L. niger* (lower panel, scale bar 200 *µ*m). Different single sections were selected for *A. sexdens* as neuropeptides occurred not in all measured sections. A representative central head section was selected for *L. niger.* For both species, neuropeptide signal intensity was visualized by false-color coding in overlay images with a low-resolution scan of the tissue sections using a viridis coloring scheme. Linear transparency was applied to make low-intensity pixels transparent. Arrows indicate single pixels with neuropeptide signals being detected; all peptide ions were shown as [M + H]^+^ (see Table 1 for *m/z* values). B) Mean MRMS mass spectrum of all measured regions as defined during the setup of the measurement for *L. niger*, insert shows tachykinin 1 ([M + H]^+^, calc. *m/z* 993.476, measured *m/z* 993.479, error 2.8 ppm). C) Mean mass spectrum for *A. sexdens*, inset shows ITG-like peptide ([M + H]^+^, calc. *m/z* 1005.5482, measured *m/z* 1005.5487, error 0.5 ppm). D, E) 3D maps of ITG-like neuropeptide and tachykinin 1 in *L. niger* (D) and *A. sexdens* (E). Data shown here are from 44 central head sections of *L. niger* and 32 sections of *A. sexdens.* The dorsoventrally cut sections were digitally stacked on each other by manual coregistration based on the low-resolution grayscale scans of the individual sections in SCiLS Lab 2023a. The section border is represented as gray line. Neuropeptide 3D distributions were visualized in volume mode using a viridis color map. Low-intensity pixels were made transparent. AST A2, allatostatin A2; MS, myosuppressin; NPLP1-4, neuropeptide-like precursor 1-derived peptide 4; sNPF, short neuropeptide F; tachykinin 1, tachykinin 4, TK-7, tachykinin-related peptide 1, 4, or 7. Orientation: P, posterior; D, dorsal.

Using spatial peptidomics of ant brains, we were able to account for a total of 8 neuropeptides “shared” between both species, namely IDLSRF-like peptide, ITG-like peptide, MS, neuropeptide-like precursor 1 (NPLP1)-derived peptides 4 and 6, sNPF, TK1 and TK4 (see Fig. 2A). To maintain a comparable measurement time to ensure sample stability to compare both species, for *L. niger*, we acquired spatially resolved mass spectra with a lateral resolution of 40-*µ*m pixel size across 44 consecutive sections, and for *A. sexdens* data from 32 sections with a pixel size of 30 *µ*m. Neuropeptide signals were not present in all measured sections, demonstrating that classical two-dimensional (2D) MALDI MSI is not sufficient to cover neuropeptide distribution, even in specimen with head sizes as small as 1 mm. High-mass resolution MRMS data enabled unambiguous assignment of *m/*z values to the neuropeptides by exact mass determination (Table 1) and is shown exemplarily for TK1 in *L. niger* (inset Fig. 2B, mass error 2.8 ppm) and ITG-like peptide in *A. sexdens* (inset Fig. 2C, mass error 0.5 ppm). This methodology illustrates the possibility to visualize hundreds of different molecules through the multiplexed MS analysis as reflected in the mean spectra of all sections for *L. niger* and *A. sexdens* (see Fig. 2B and C).

We used spatial clustering of the full MSI data including all signals from peptides to lipids to group the molecular images after spatial and spectral similarities (Fig. S2). The spatial clusters showed a clear separation between the local chemistry of tissues surrounding the brain (i.e. musculature) and the brain itself, as verified through overlays with optical scans of the same sections (Fig. S2). All detected neuropeptides were centered in the brain-correlated spatial cluster indicating that each 3D MSI data set contained the in-depth neuropeptidomic composition of the ants’ brains (see Fig. 2D and E for ITG-like peptide and TK1 for *L. niger* and *A. sexdens*). However, without precise knowledge on the 3D orientation and colocalization between peptides and specific regions of the brain, analysis and interpretation of the different neuropeptide distributions remained challenging. To further exploit the results of the 3D MSI data, we wanted to allocate the 3D neuropeptide chemistry to well-studied (sub)regions of the ants’ brains, such as antennal and optical lobes or mushroom bodies (MB).

### Anatomical model of ant brains based on µCT data

Using noninvasive µCT, we generated micrometer-scale models of two ant heads and their interior structures from developmental stages and castes of *L. niger* and *A. sexdens* similar to the samples used for spatial peptidomics. These high-resolution anatomical µCT models provided the 3D framework to precisely coregister the serial MSI data.

**Fig. 3.**
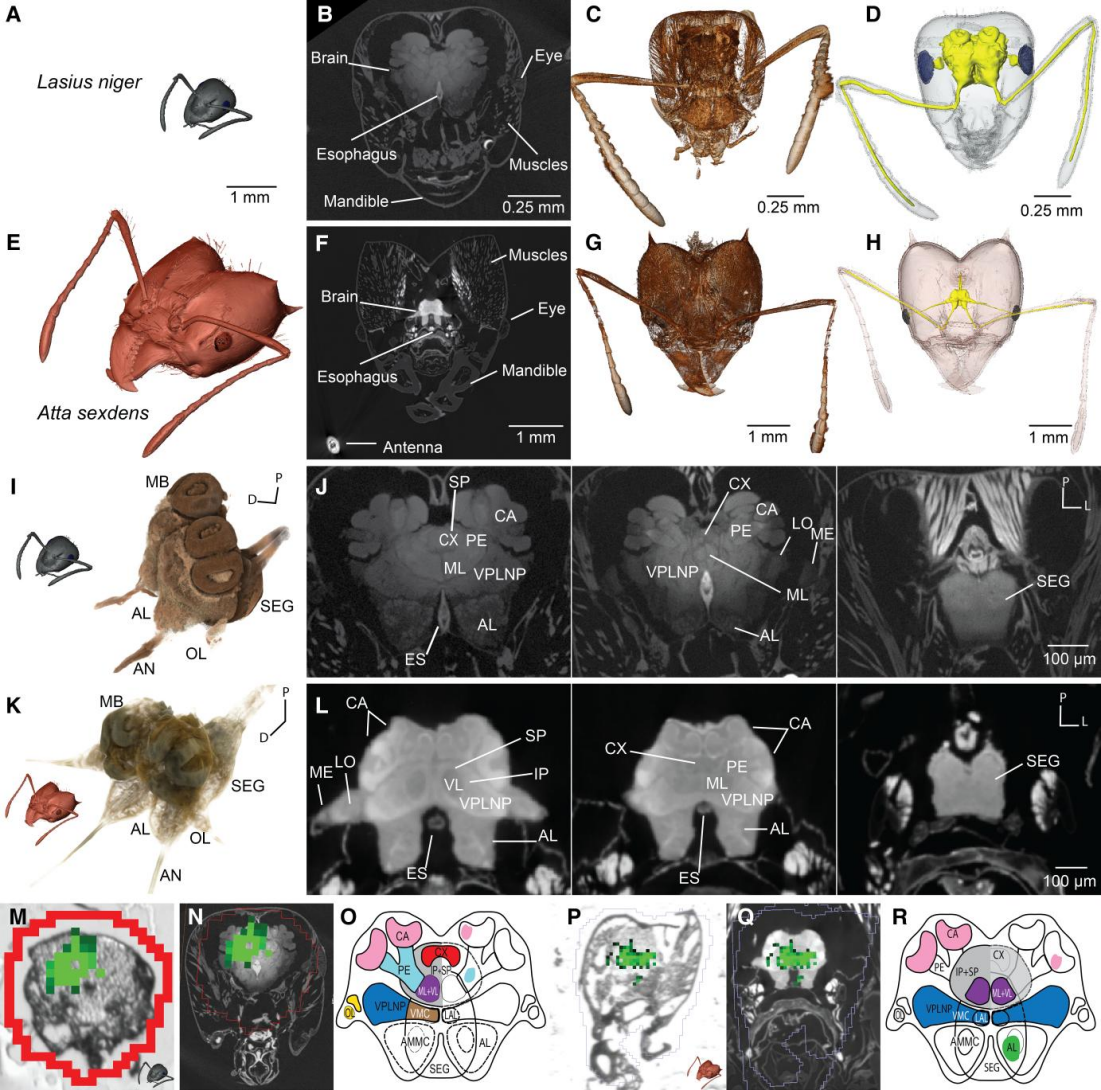
3D head anatomy of *L. niger* and *A. sexdens* with neuropeptide TK1 localization. A–H) Anatomic proportions of brain to head ratios between *L. niger* (A–D) and *A. sexdens* (E–F). A, E) Comparable scale of two µCT-based 3D models of two worker castes; B, F) virtual plane of transverse sections through the 3D µCT volume; C, G) volume renderings (Drishti software version 2.6.4); D, H) surface reconstructions of head capsule, brain, antennal nerves, and eyes (AMIRA software version 6.3). I–J) Detailed brain anatomy resolved for *L. niger* (I, G) and *A. sexdens* (K, L) using µCT. I, K) Volume renderings of both brains; G, L) virtual planes of transverse sections through the 3D µCT volume. M–R) Coregistration of MALDI MSI ion map of neuropeptide TK1 for *L. niger* (M–O) and *A. sexdens* (P–R). M, P) Ion map on a tissue section of TK1; N, Q) overlay of MALDI MRMS MS and 2D µCT virtual slice; O, R) brain drawing of the TK1 distribution of the tissue sections shown in M) and P), respectively. AL, antennal lobe; AMMC, antennal mechanosensory and motor center; AN, antennal nerve; CX, central complex; ES, esophagus; IP, inferior protocerebrum; LAL, lateral accessory lobe; MB, mushroom bodies (CA, calyx; ML, medial lobe; PE, pedunculus; VL, ventral lobe); OL, optical lobe (LO, lobula; ME, medulla); SEG, subesophageal ganglion; SP, superior protocerebrum; VMC, ventromedial cerebrum; VPLNP, ventro-posterolateral neuropils. Orientation: D, dorsal; L, lateral; P, posterior.

For µCT sample preparation, we adapted a combination of protocols using phosphotungstic acid (35, 51) and added subsequent critical point drying (CPD) (37). CPD is conventionally used to preserve tissues of small arthropods for µCT scanning for an improved signal-to-noise (s/n) ratio as liquids from nontissue-filled compartments around the brain are removed. Phosphotungstic acid staining in combination with CPD enabled us to scan the intact heads of both ant species and resolve substructures of the ant brain down to a submicron level (see Fig. 3).

Particularly challenging was to sufficiently contrast the brain and its substructures inside the intact head capsule, as the impermeable head capsule caused a blockage of the contrasting agents. Structures such as protective exoskeletons or shells present a persisting problem for staining solutions and are conventionally removed when working with arthropods or other invertebrate organisms (21). Notably, microdissection of such small specimens is time-consuming, requires a high level of skill, and can induce distortions up to major damage of the delicate micrometer-scale anatomy. Therefore, we severed the head from the body, allowing the phosphotungstic acid solution to diffuse directly through the nerve cord into the brain (Fig. S3). Using this procedure, we were able to obtain very small volume pixel (voxel) sizes (*L. niger*: 0.805 *µ*m; *A. sexdens*: 3.111 *µ*m) at sufficient contrast and s/n to resolve fine structures of both brains. Moreover, not only brain and subesophageal ganglion (SEG) were contrasted, but also off-branching nerves into antennae, eyes, mandibulae, and maxillae of both species also showed high levels of contrast. Structures of different materials, less rich in lipids, such as musculature or cuticle were not penetrated equally strong by the contrasting solution. The strong contrasting of the nervous tissues enabled us to semiautomatically segment the brain and its surrounding tissues for subsequent 3D surface reconstructions (see Fig. 3).

The ant brain follows the general design of insect brains and, as in other holometabolous insects, is fused to the SEG (52). With the µCT data set of each species, both high-resolution 3D anatomical models enabled us to navigate through the ant brains and the SEG to identify the organ's subdivision. Following the nomenclature for brain proposed by Ito et al. (53) and Bressan et al. (54), we recognized 20 brain compartments in the µCT data set of *L. niger* and 16 in *A. sexdens*, including optic lobes (OL), MB, central complex (CX), antennal lobes (AL), superior and inferior protocerebrum (SP + IP), antennal mechanosensory and motor center (AMMC), lateral accessory lobe (LAL), ventroposterolateral neuropils (VPLNP), ventromedial cerebrum (VMC), and also the SEG in both species (see Fig. 3I–L). Due to the almost 4-fold increased magnification for *L. niger* compared with the *A. sexdens* head, we could resolve more neuropils in *L. niger*, for instance lamina in OL and central body upper and lower units, protocerebral bridge, and noduli in the CX (Table S3).

To date, ant brain neuropil maps were established by using confocal laser scanning microscopy (54, 55). Although this technique provides subcellular resolution and cell-specific staining, µCT does not require dissection of the brain, immunostaining followed by extensive imaging times, and is therefore considerably less time-consuming. For instance, due to the specificity of the antibodies for synapsis detection, the brain map built by Bressan et al. (54) resulted in a highly detailed structure of the axon fascicles associated with compartment boundaries. Most of these structures were not observable in the here generated µCT atlas. However, methodologies have been developed to combine antibody labeling with heavy metal contrasting for generating a tissue-specific contrast detectable with µCT (36). Today, the increased imaging and processing speeds of high-resolution µCT provide a tool to quantitatively compare the neuroanatomy of many individuals across different developmental stages and castes to screen for heterogeneities in the plasticity of the brain.

For confocal and/or laser scanning microscopy, the in-depth resolution (*z*-axis) is optically limited to about half of its lateral resolution (*y*- and *x*-plane). This results in unequally shaped (anisotropic) voxels, 3D “volume pixels,” whereas µCT provides the same voxel edge length along all axes (isotropic voxels) (56). This allows for the calculation of distortion-free biovolumes that without interpolation allow for quantitative analyses. Both ant brain atlases generated here by µCT display the anatomical relationships between the brain, musculature, and head capsule including mandibles and antennae (see Fig. 3). Additional optical images of cryo-sections from snap-frozen samples served as controls for shrinking artifacts from the alcohol-based µCT contrasting protocol.

Despite nearly two orders of magnitude difference in head volume (*L. niger*: 0.031 mm^3^; and *A. sexdens*: 1.38 mm^3^), the brain of *L. niger* is only half the volume of *A. sexdens* (*L. niger*: 0.026 mm^3^ and *A. sexdens*: 0.052 mm^3^) (see Fig. 3). However, the ratio of brain volume as compared with the overall head volume was much higher in *L. niger* than that in *A. sexdens*, revealing that the brain of *L. niger* consumes ∼80% of the head capsule, whereas in *A. sexdens*, only ∼7% of the head volume was occupied by the brain. These precise volumetric measurements provided valuable information when investigating the relationships between size and function of the brains. For example, once the distinct localization of a neuropeptide is known, quantitative LC–MS/MS in combination with µCT could in future studies allow for calculating the neuropeptide concentrations in each (sub)region of the brain and a comparison between different castes. Complementing the 3D microstructure in correlation to the 3D neuropeptide chemistry of the brain could shed light on how morphological adaptations, such as miniaturization, relate to behavior of ants and other social insects (57).

### Integrative spatial peptidomics and anatomy shows brain region-specific chemistry

**Fig. 4.**
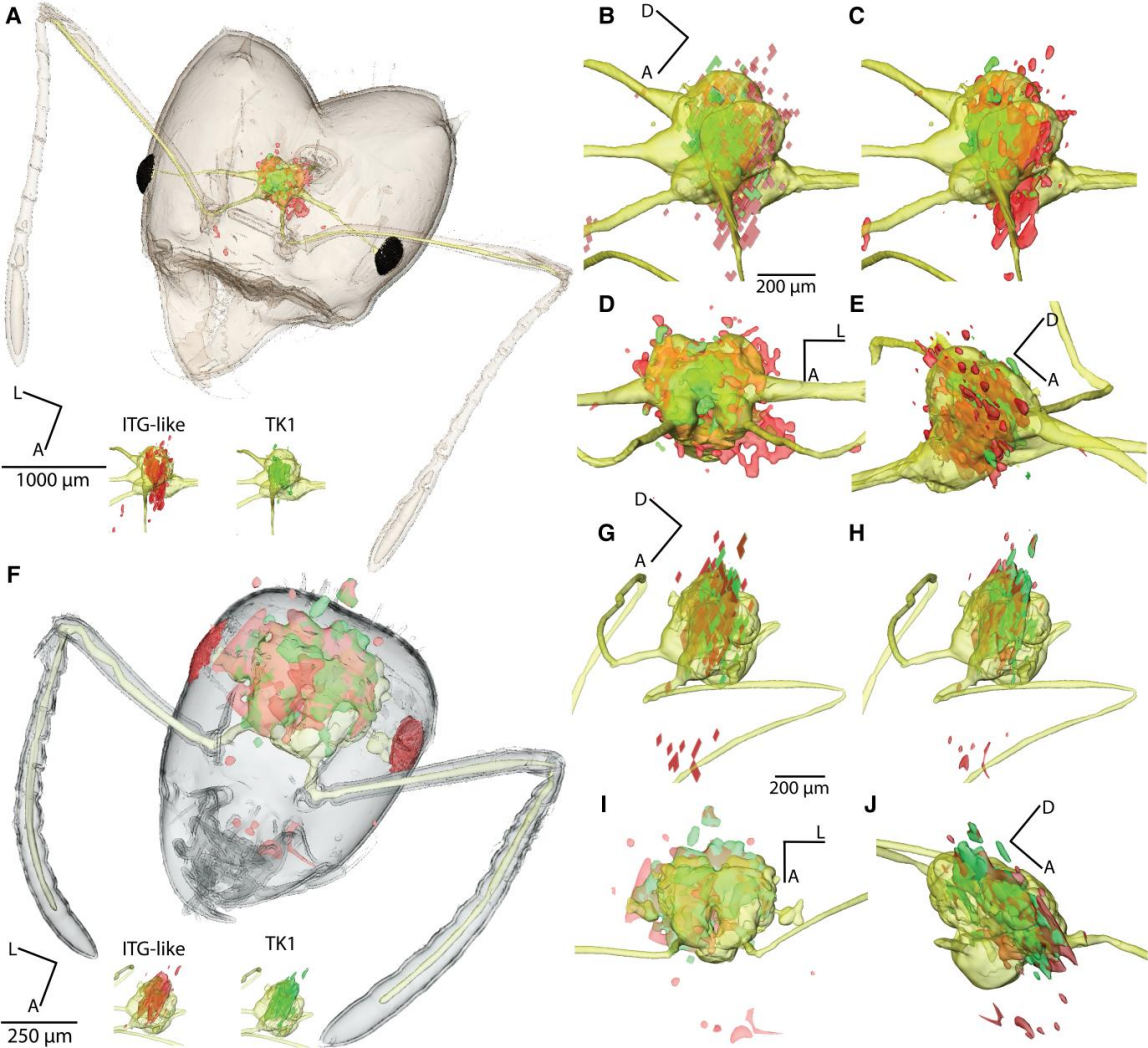
Integration of 3D neuropeptide distributions with microanatomy reconstructed 3D data. Surface renderings of segmented head capsule, brain/nerves, and coregistered 3D distributions of TK1 and ITG-like neuropeptide in *A. sexdens* (A) and *L. niger* (F). C–J) Panels with four representations for surface reconstructions of the brain and both neuropeptide distributions of each species (*A. sexdens*, B–E; and *L. niger*, G–J); dorso lateral view, no smoothing and interpolation (top-left), dorso lateral view, smoothing and interpolation (top-right), dorsal view (bottom-left), and latero-ventral view (bottom-right).

Integrating the serial spatial peptidomics maps into the anatomic µCT-generated models resulted in 3D neuropeptide atlases of *L. niger* and *A. sexdens.* We visualized the coregistration between the 3D distributions of TK1, ITG-like neuropeptide, and the µCT-based surface reconstructions of *L. niger* and *A. sexdens* (see Fig. 4).

To create a reliable 3D coregistration, we based the matching between MSI and µCT data on manual annotations by experts in neuro- and invertebrate anatomy. The bright-field microscopy images of the tissue sections, used for MSI, provided key landmarks to determine in which orientation the chemical images had to be coregistered into the digital µCT data sets. There are three challenges for coregistering MSI data with other imaging modalities (26). Firstly, chemical distributions of analytes recorded by MSI must not necessarily correlate with a distinct anatomy of a tissue or organ. Secondly, MSI sensitivity for ion detection, the number of molecules that need to be ionized per pixel, limits MSI spatial resolutions to one or two orders of magnitude lower than radiation-based techniques, such as light microscopy (58). Thirdly, MSI is a surface-scanning technique that requires physical sectioning of frozen or chemically fixed tissues, leading to distortions and subcellular damage of the tissue. Therefore, we based our coregistration between MSI and µCT on the optical images of the thaw-mounted cryo-sections that were recorded with a low-resolution slide scanner. Due to the generally limited quality of slide scanner images, subregions of the brain were difficult to identify. Notably, we chose to use a slide scanner over an automated microscope, because it provided the fastest way to record the whole section series as swiftly as possible between cryo-sectioning and MSI to prevent oxidation of the lipid-rich brain tissue. For the coregistration, distinct tissue features and their relation to each other such as eyes, head capsule, esophagus, SEG, and MB served as reference points for a precise alignment of optical images, used for MSI and the corresponding µCT image plane (see Figs. 3 and S4). Critical for our workflow was to ensure that the correct proportions of the coregistered volumes, the *z*-distances between the individual tissue sections were documented during sectioning and scaled proportionally during coregistration with µCT data (Fig. S4). In a last step, once the optical images were individually matched to their corresponding virtual µCT image plane (see Fig. S4), their coordinates in the 3D space could be transformed to the MSI neuropeptide maps and visualized as 3D volumes (see Fig. 4).

Although different animals for both MSI and µCT were used, we chose comparable castes and sizes to better correlate neuropeptide distributions with ant brain anatomy. The coregistration of chemical and anatomic data enabled us to calculate volumetric relations of brain size, relative amounts of neuropeptide and overall size of the head. For instance, TK1 and ITG-like neuropeptide occur in similar regions throughout the brains of *L. niger* and *A. sexdens*. Despite the substantially different anatomy and lifestyle of both ant species, their brain size is within the same order of magnitude, and the distribution of essential neuropeptides, such as TK1 and ITG-like peptide, correlates between both species. Although conclusions from this comparison will have to be tested quantitatively, our comparative snapshot could help us to determine conserved and distinct features of the neurometabolism between ant species. These observations are in line with previous MS analysis of neuropeptides at single-cell resolution (59).

Even though MSI imaging provides an unprecedented view of neuropeptide distributions at anatomic resolution, exploration of the magnitude of chemistry detected has to be done. We provide the data sets, ready to browse online, for lipids and many other nonpeptide metabolites in www.metaspace2020.eu (ant brain) as a community resource for further insect brain chemistry questions.

### Spatial peptidomics reveals how neuropeptide distributions differ in ant brains

The region-specific localization of ten neuropeptides in both species strongly varied (Table 2). Consequently, we cannot exclude the presence of low neuropeptide levels in other regions of the brain. However, both species were measured with comparable settings allowing us to compare the spatial distribution of relative abundances of neuropeptides otherwise impossible with label-free omic approaches. In general, neuropeptides of *L. niger* showed wider distributions compared with *A. sexdens*. This could reflect *L. niger* being a species with only one morphological caste of workers with less specialization than in *A. sexdens* where at least six different kinds of specialized workers operate delimited tasks (60). Regarding the occurrence of the neuropeptides in different brain regions, TK1, ITG-like, and sNPF appeared widely distributed in the neuronal organ, exhibiting a similar brain neuropil allocation in both species (see Table 2 and Fig. 4). Similarly, NPLP1-4 and NPLP1-6 were detected across most regions of the brain of *L. niger*, but only in the ventroposterolateral neutropil region of *A. sexdens*. TK4 and IDLSRF-like peptide were imaged in half of the brain regions annotated for *A. sexdens* and found in most regions across the brain of *L. niger*. The coregistration between both techniques that were applied to different individuals restricted an allocation of neuropeptides to the level of the annotated brain regions. However, the confidence of identification and coregistration per brain region was increased due to our 3D approach, as we could trace each region throughout the consecutive sections of our serial brain slices.

**Table 2.** Distribution of the common neuropeptides detected and identified from brain tissue sections via spatial peptidomics after coregistration with µCT data set in *L. niger* and *A. sexdens*.

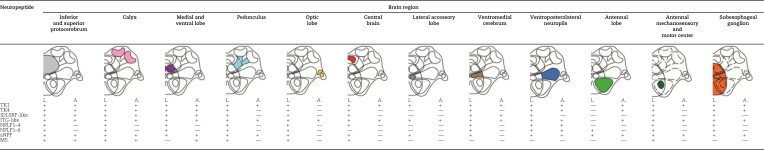

+, presence; —, absence; *L.*, *Lasius niger*; *A.*, *Atta sexdens*. Neuropeptides: IDLSRF-like peptide; ITG-like peptide; MS, myosuppressin; NPLP1-4 and NPLP1-6, neuropeptide-like precursor 1-derived peptides 4 and 6; sNPF, short neuropeptide F; TK1 and TK4, tachykinin-related peptides 1 and 4; colours indicate the different brain regions.

How neuropeptide distributions, for example left–right asymmetries, regulate each other and relate to neurobiological processes such as lateralization and ultimately behavior remains to be determined. Considering that neuropeptide distributions vary along the animals’ lifecycles, these changes can be very fast for some developmental stages. For instance, in the honeybee (*Apis mellifera*), two neuropeptides change significantly during the first 25 days of the adult stage (19). Validating our findings in a biological context of neuropeptide function (Table S4) through subsequent studies of larger cohorts with animals of different castes and developmental stages could reveal how spatial heterogeneity of neuropeptides in correlation to microanatomic brain plasticity shapes social behavior in ants and other insects.

### Atlases with multiomic data are emerging tools for organismal biology

Applications of multiomic 3D atlases that combine anatomic structure with spatial proteomic, transcriptomic, or metabolomic data have become an emerging field (25, 26, 61, 62). These modern multiomic atlases build upon the extensive knowledge generated from large-scale descriptive studies that for instance mapped the 3D ultrastructure of a whole fly brain (63), or the early human development imaged by light-sheet microscopy (64), by adding a layer of functional data—a combination of structure and function. Still, despite rapid improvements on the technological side in terms of imaging speed, spatial resolution, data processing, and cost (16), there are still major challenges making large-scale correlative studies difficult. One limiting factor can be access to equipment and expertise in imaging and data processing which either is often cost intensive or requires interdisciplinary collaboration. Notably, X-ray microtomography setups and MALDI-MSI equipment including processing software have been acquired by labs and core facilities at most large research institutions through public funding and should be accessible to the broader scientific community within collaborative efforts.

Harmonization of sample processing workflows for both 3D imaging techniques, the correlative analysis between modalities, remains the most critical step. Coregistration (65, 66) of the different types of multiomic imaging data and the correlative analysis across modalities (67) has not yet been integrated into a user-friendly software with a graphical user interface. Spatial omic techniques, such as MSI, are often multiplexed and enable the detection of hundreds to thousands of signals at once without prior knowledge of the sample. Although the data are more complex and the analysis challenging, untargeted analysis comes with the unique advantage that it allows for the discovery of hitherto unknown biological mechanisms if known anatomic or cellular structures are correlated to spatial chemistry (68, 69).

In this study, we highlight the power of spatial peptidomics for deciphering ant neurochemistry. The provided insights into key signaling molecules ultimately allows for identification of factors that drive caste identity and behavior. Our approach offers an integrative 3D visualization of the native, label-free biochemistry with undisturbed microanatomy allowing for comparisons of neuropeptide levels across the different subregions of the brains of two ant species. Our atlas contributes to a better understanding of the processes and interactions between signaling molecules in the brains of ants and other insects that have allowed for their high level of coordination to not only thrive in nearly every ecosystem but to become essential for ecosystem health.

## Material and methods

### Specimen

Workers of the monomorphic black garden ant *L. niger* were taken from laboratory colony maintained in an incubator in summer conditions (14-h light, 27°C/10-h darkness, 20°C; 60% humidity) and fed ad libitum. Medium and large castes of polymorphic leafcutter ant *A. sexdens* were collected from Schönbrunn Zoo colony (Vienna, Austria).

### Genome search and mass calculation of neuropeptides

Published neuropeptide precursor sequences of *A. echinatior* (44) and *C. floridanus* (45) were used to query the NCBI Whole-Genome Shotgun (WGS) database of *A. cephalotes* (project number PRJNA279976) (47) and *L. niger* (project number PRJNA269328) (46) via tBLASTn online using BLOSUM62 matrix. Hits with the lowest *E*-values were manually annotated and the peptide sequence was determined according to the obtained alignment of the query and the subject. Peptide masses were calculated using Peptide Mass Calculator v3.2 online tool (http://rna.rega.kuleuven.be/masspec/pepcalc.htm).

### RNA isolation and sequencing of MS, TK, AST, and sNPF precursors

RNA extraction of 2–5 heads of *L. niger* or *A. sexdens* was done using Quick-RNA MiniPrep kit (Zymo Research). Samples were homogenized in 350 *μ*l Lysis Buffer with 2–4 BashingBead (Zymo Research) using homogenizer Precellys 24 (Peqlab) for 3 × 30 s 6,000 rpm. DNase I treatment was performed after eluting RNA from columns prior to reverse transcription accordingly: samples (50 *μ*l) in 0.5 × DNase I buffer (Thermo Scientific, #B43) were incubated with 1 U of DNase I for 20 min at room temperature following the inactivation of DNase I by adding EDTA to 2.5 mm final concentration and heat denaturation at 70°C for 10 min. Reverse transcription was performed using High-Capacity cDNA Reverse Transcription Kit (Applied Biosystems) according to manufacturer's instructions. PCR products of precursor sequences were obtained via standard PCR using Phusion Hot Start II DNA Polymerase (Thermo Scientific) and primers (Sigma-Aldrich) (Table S5). The PCR fragments were cut from an agarose gel, purified using GeneJET Gel Extraction kit (Thermo Fisher Scientific) and sequenced at LGC Genomics (Berlin, Germany) using both reverse and forward primers. Using standard PCR, we obtained only the beginning (∼700 bp) of *A. sexdens* TK precursor, while the end was confirmed via Rapid Amplification of 3′ cDNA Ends (3′ RACE) technology: reverse transcription was performed using 3′-Race-RT-primer, first PCR was done using 3′Race-PCR-Rev and A.sex-TK-Fw primers, and then the product of nested PCR (361 bp), obtained using 3′-Race-PCR-Rev and 3′-Race-PCR-Fw primers and diluted reaction of first PCR as a template, was sequenced. The confirmed *A. sexdens* TK precursor sequence is much shorter compared with *A. cephalotes* or *A. echinatior* (Fig. S1). The sequence of *L. niger* TK precursor was confirmed by obtaining and sequencing two overlapping PCR products using primers L.nig-TK-Fw1 + L.nig-TK-Rev1 for the N-termini and L.nig-TK-Fw2+ L.nig-TK-Rev2 for C-termini.

### MALDI MRMS imaging and data analysis

Animals were placed in a reaction tube and killed by immersion in liquid nitrogen for 5 s (*L. niger*) or by freezing at −80°C (*A. sexdens*) and decapitated under the stereomicroscope. Heads were immersed in a mold with 10% gelatine (cold water fish skin, SIGMA 67041) to obtain a block suitable for cryo-sectioning at 20°C (Leica CM1950). Dorsoventral serial sections of 10 *µ*m were mounted onto conductive indium-tin oxide (ITO)-coated glass slides (Bruker Daltonics GmbH & Co KG, Bremen, Germany).

Slides were dried under vacuum for 15 min and then coated with alpha-cyano-4-hydroxycinnamic acid (HCCA) using an ImagePrep device (Bruker Daltonics, Bremen, Germany). HCCA matrix [7 mg/ml dissolved in 50% (*v*/*v*) acetonitrile, 0.2% (*v*/*v*) trifluoroacetic acid] was sprayed with the ImagePrep default program for this matrix in five phases. In the first initialization phase, matrix was applied in 10 cycles with a fixed spray time of 2.5 s, spray power set to 15, modulation 40, incubation time 15 s, and dry time 65 s. In phases 2–5, spray time and dry time were automatically controlled by the instruments integrated scattered light sensor. The spray power was set to 25, modulation was 45, and incubation time was 30 s.

Data were acquired on a Bruker solariX XR MRMS instrument (Bruker Daltonics, Bremen, Germany) equipped with a 7T superconducting magnet, MALDI ion source, and smartbeam II laser technology. Compass ftmsControl was used to set the instrument parameters. A total of 1,000 laser shots were collected per spectrum in the mass range of 300–3,000 Da (*L. niger*) or 300–2,600 Da (*A. sexdens*). The raster size was set to 30 (*A. sexdens*) or 40 *µ*m (*L. niger*) resulting in data sets containing 143,359 spectra or 25,707 spectra, respectively. Mass calibration was performed externally on sodium trifluoroacetate in electrospray ionization mode. The mass resolution was >220,000 at *m/z* 400. FlexImaging Software version 4.1 was used to define the measurement areas. Raw data for each ant were imported to the software SCiLS Lab version 2023a (Bruker Daltonics, Bremen, Germany) and normalized to total ion count (TIC). For the 3D MALDI model, low-resolution scan images were stacked virtually in *z*-direction according to the section outline with 10-*µ*m distance using the SCiLS Lab registrator. Neuropeptide distributions in 2D and 3D were visualized by false-color coding, and low-intensity was were made transparent.

### µCT scanning

Specimens were placed 30 s in a petri dish on ice before decapitation with a sharp razor blade. The heads were fixed in Bouin's solution overnight. Then, the fixative was removed with disodium hydrogen phosphate buffer (0.1 m, pH 7.2, 1.8% sucrose) by 2 × 30 min washes, followed by an overnight immersion in the same buffer and 2 × 30 min washes the next day. Afterwards, the heads were dehydrated in a graded ethanol series and contrasted for 2 weeks using a solution of 1% phosphotungstic acid in 70% ethanol, submersed in acetone and CPD using a Leica EM CPD300 (Leica Microsystems GmbH, Wetzlar, Germany). Contrasting and CPD protocols were adapted from previous studies (34, 35, 37).

After CPD, the heads were carefully mounted on the tip of a glass syringe using a hot glue gun. A nanotom m (GE Measurement & Control, Wunstorf, Germany), equipped with an X-ray cone beam setup was used for scanning. Magnifications were defined through geometrical relations that were based on the distances between sample, source, and detector. The heads were scanned at a voltage of 110 kV, 120 *μ*A current, and 0.75-s exposure time at an averaging of 4, which resulted in a scanning time of 90 min and 1,500 number of projections acquired during scan. The tomographic reconstruction was performed using the standard parameters offered by the phoenix datos|x 2.2 reconstruction software on a separate workstation. *L. niger*: resulted in a voxel size of 0.805 *µ*m and *A. sexdens*: 3.111 *µ*m). Being able to move the smaller head of *L. niger* closer to the X-ray source and further away from the detector resulted in a higher magnification due to the X-ray cone beam. For feasible data processing, the 16-bit volume was cropped, converted to 8-bit depth, and formatted to the *.hdr format in the 3D visualization software VGStudio (2.2).

### 3D visualization of µCT-derived anatomy

3D volume exploration and rendering were done with the v3D volume exploration and visualization software Drishti, version 2.5.1 (70) and Amira 6.3 (Thermo Fischer Scientific, USA). Segmentation and surface reconstruction of specific anatomical structures such as head capsule, eyes, brain, and nerve branches were done in Amira v.6.3 in an adapted workflow after Ruthensteiner et al. (71).

### Coregistration of µCT and MALDI-MSI data

MALDI-MSI and µCT data sets were combined in 3D space using the visualization software Amira v.6.3 (FEI Co., USA). The coregistration workflow had to be adapted as both MALDI-MSI and µCT data sets originated from different specimens and imaging techniques. Because the ant heads are heterogeneous tissue samples and were neither chemically fixed nor infiltrated with embedding medium, the cryo-sections showed strong distortions and ruptures. Therefore, orientation and subsequent fitting of the optical image stack into the µCT volume were based on visually comparable structures such as eyes, brain regions, and mouthparts.

For the coregistration, the SCiLS aligned image stacks of the data were used. The general coregistration workflow consisted of two main steps. In the first step, the series of optical images of the MALDI-MSI sections was coregistered to the µCT data. In the second step, the same transformation parameters were then applied to the image stacks of the ion maps of TK1 and ITG-like neuropeptide. The same workflow was used for both ant species.

Initially, the *z*-distances between the optical images had to be adjusted because not every section was used for MALDI-MSI. Gaps in the image stacks were filled with empty images. The optical image series of the tissue sections with the correct spacing was then imported into AMIRA. The corresponding sectioning planes between the optical images and µCT slices could be determined because the µCT volume was virtually sliced along each of the *xyz*-planes. With the series of optical images being aligned and the sectioning plane determined, the volume of the optical images was fitted into the 3D space of the µCT volume through manual translation, rotation, and proportional scaling by aligning anatomical structures.

The ion image stacks of TK1 and ITG-like neuropeptide were then coregistered into the volume, using the same transformation parameters, determined with the optical image series. For visualization purposes, ion distribution stacks were also threshold segmented and visualized as surfaces in the 3D model. The surface reconstructions allowed for simultaneous visualization of the ion maps in the 3D model based on different transparency settings.

## Supplementary Material

pgad144_Supplementary_Data

## Data Availability

All microscopy and µCT data sets can be directly downloaded from Figshare: *A. sexdens* (https://doi.org/10.6084/m9.figshare.21482895.v1) and *L. niger* (https://doi.org/10.6084/m9.figshare.21482898.v1). MALDI-MSI data were deposited within a project on the www.metaspace2020.eu database (https://metaspace2020.eu/project/antbrain_AttaSexdens; https://metaspace2020.eu/project/antbrain_Lasiusniger) and can be browsed online for lipids and other metabolite distributions.
