# Supplementary material for: Multiplexed neuropeptide mapping in ant brains integrating microtomography and three-dimensional mass spectrometry imaging
Source: PNAS Nexus. 2023 Apr 25;2(5):pgad144. doi: 10.1093/pnasnexus/pgad144 (PMC10194420; doi:10.1093/pnasnexus/pgad144)
Supplement: pgad144_Supplementary_Data [file pgad144_supplementary_data.pdf]

Supplementary Information

Multiplexed neuropeptide mapping in ant brains integrating microtomography and three-dimensional mass spectrometry imaging

Benedikt Geier[a,b†], Esther Gil-Mansilla[c†], Zita Liutkeviciute[c], Roland Hellinger[c], Jozef Vanden Broeck[d], Janina Oetjen[e,f*], Manuel Liebeke[a,g*] and Christian W. Gruber[c*]

(a) Department of Symbiosis, Max Planck Institute for Marine Microbiology, Bremen, Germany
(b) Department of Pediatrics and Infectious Diseases, Stanford School of Medicine, CA, USA
(c) Center for Physiology and Pharmacology, Medical University of Vienna, Vienna, Austria
(d) Molecular Developmental Physiology and Signal Transduction Group, Zoological Institute, KU Leuven, Leuven, Belgium
(e) Bruker Daltonics GmbH & Co. KG, Bremen, Germany
(f) MALDI Imaging Lab, University of Bremen, Bremen, Germany
(g) Department of Metabolomics, Institute of Human Nutrition and Food Science, Kiel University, Kiel, Germany

[†] these authors contributed equally to this work

[*]*Corresponding author:* Correspondence and requests for materials should be addressed to J.O., M.L. or C.W.G. (email: janina.oetjen@bruker.com, mliebeke@mpi-bremen.de, christian.w.gruber@meduniwien.ac.at)

This PDF file includes:

Figures S1 to S4

Tables S1 to S5

Data S1

Supplementary References

```
L.niger         MLINSVLFLAVWISSSLAEELSPNDATSAKRAPMGFQGMRGKKNLIPT--EHNKLSKRTL
A.echinator     MLFSSVLFLAVWTSSSFAEES-SNDAASAKRAPMGFQGMRGKKDLIPTIAEHNELSKRTL
A.sexdens       ----SVLFLAVWTSSSFAEES-SNDAASAKRAPMGFQGMRGKKDLIPTVAEHNELSKRTL
A.cephalotes    MLFSSVLFLAVWTSSSFAEES-SNDAASAKRAPMGFQGMRGKKDLIPTVAEHNELSKRTL
                    ******** ***:***   ***:****************:****  ***:******

L.niger         MDFQDTRDNKDSNAPDIEDNLLHDEFDKRAPMGFQGMRGKKDYLMPDFEDSYFRD--EKR
A.echinator     VNFQDK----DSSASEIEDNLLHDEFDKRAPKGFQGMRGKKDYLTPDFEDSYFLEDYNKR
A.sexdens       VNFQDK----DSSASEIEDNLLHDEFDKRAPKGFQGMRGKKDYLIPDFEDSYFLEDYDKR
A.cephalotes    VNFQDK----VSSASEIEDNLLHDEFAKRAPKGFQGMRGKKDYLIPDFEDSYFLEDYDKR
                ::***.     *.* :********** **** ************ ******** :  :**

L.niger         APMGFQGMRGKKIVSDDEYYKRAPMGFQGMRGKKSSEEVLDEIEKKA-AMGFYGTRGKKT
A.echinator     APMGFQGMRGKKAILEDEYYKRAPMGFQGMRGKKSLEEMLSEIEKRA-ALGFYGTRGKKT
A.sexdens       APMGFQGMRGKKAILEDEYYKRAPMGFQGMRGKKSLEEVLSEIEKRA-ALGFYGTRGKKT
A.cephalotes    APMGFQGMRGKKAILEDEYYKRAPMGFQGMRGKKSLEEVLSEIEKRAASLGFYGTRGKKT
                ************ : :******************* **:*.****:* ::**********

L.niger         YVLEYPEDYEKRLLAMGFQGIRGKLKEFPVEWEKRAPMGFQGMRGKKALLDEIEELEKRT
A.echinator     YVFEYPQDYEKRLLAMDFQDMHNKIK---EEWEKRAPMGFQGMRGKKALYDEIEELEKRA
A.sexdens       YIFEYPQDYEKRLLAMEFQDMHNKIK---EEWEKRASMGFQGMRGKKALYDEIEELEKRT
A.cephalotes    YIFEYPQDYEKRLLAMEFQDMHNKIK---EEWEKRASMGFQGMRGKKALYDEIEELEKRT
                *::***:********* **.::.*:*    ****** ************ *********:

L.niger         VMGFQGMRGKKNAFENYVDYYM-DPDMDFDKRAPMGFQGMRGKKDSDKRAPMGFQGMRGK
A.echinator     LMGFQGMRGKKDGFENYIDYYIDDPDMDFDKRAPMSFQGMRGKKDTDKRAPMGFQGMRGK
A.sexdens       LMGFQGMRGKKDGFENYIDYYIDDPDMDFDKKASMSFQGMRGKKDTDKRAPMGFQGMRGK
A.cephalotes    LMGFQGMRGKKDGFENYIDYYIDDPDMDFDKRASMSFQGMRGKKDTDKRAPMGFQGMRGK
                :**********:.****:***: ********:* *.*********:**************

L.niger         RNTGQRFDTGMDFETRTSNEYQGTSNRRNALASCQLEKRSPFRYFEMRGKKNPRWELRGM
A.echinator     RSVGQRFDPNMNFG--SLNEYQGRSNRRHILASCQVEKRSPFRYFEMRGKKNPRWELRGM
A.sexdens       RSVAQRFEPSMNFG--PLNEYQDSQ#----------------------------------
A.cephalotes    RSVGQRFEPSTNFG--PLNEYQGMGNRRHILASCQVEKRSPFRYFEMRGKKNPRWELRGM
                *...***: . :*     ****.

L.niger         FVGVRGKKWATAPYEDDSPFISVFDNTERIGVDGDSPAILDSQ-----------------
A.echinator     FVGVRGKKWANAPYEDNSPFIRVLDNTERIGMDGDSPTTLGNRLADSQLISNIYVAVLFI
A.sexdens       ------------------------------------------------------------
A.cephalotes    FVGVRGKKWANAPYEDNSPFIRVLDNTERIGMDGDSPTTLGNRLADSQLVYRPERYDLDH

L.niger         ------------------------------------------------------------
A.echinator     TSFIFH---------VYLMAKYACSIYQVSSNLSERRERKINFFSFHTNVQNIFFSAFYM
A.sexdens       ------------------------------------------------------------
A.cephalotes    PSGYWNKPKLQNEPMINNGASAVCSVFVIVSTVD--------------------------

L.niger         ---------
A.echinator     QCIIFKNLC
A.sexdens       ---------
A.cephalotes    ---------
```

Supplementary Figure S1. **Alignment of tachykinin-related peptide (TK) precursors of *A. sexdens*, *A. cephalotes*, *A. echinator* and *L. niger*.** *A. sexdens* TK7 was confirmed to be a single amino acid shorter compared to *A. cephalotes* (shown in light blue). The cloned *A. sexdens* TK precursor is shorter (stop codon indicated by #; using 3' RACE and poly dT primer, see Suppl. Table S3) as compared to the other species lacking two neuropeptides (highlighted in grey, TK5 and TK6 according to the Suppl. Table S1); it needs to be confirmed whether a longer version co-exists.

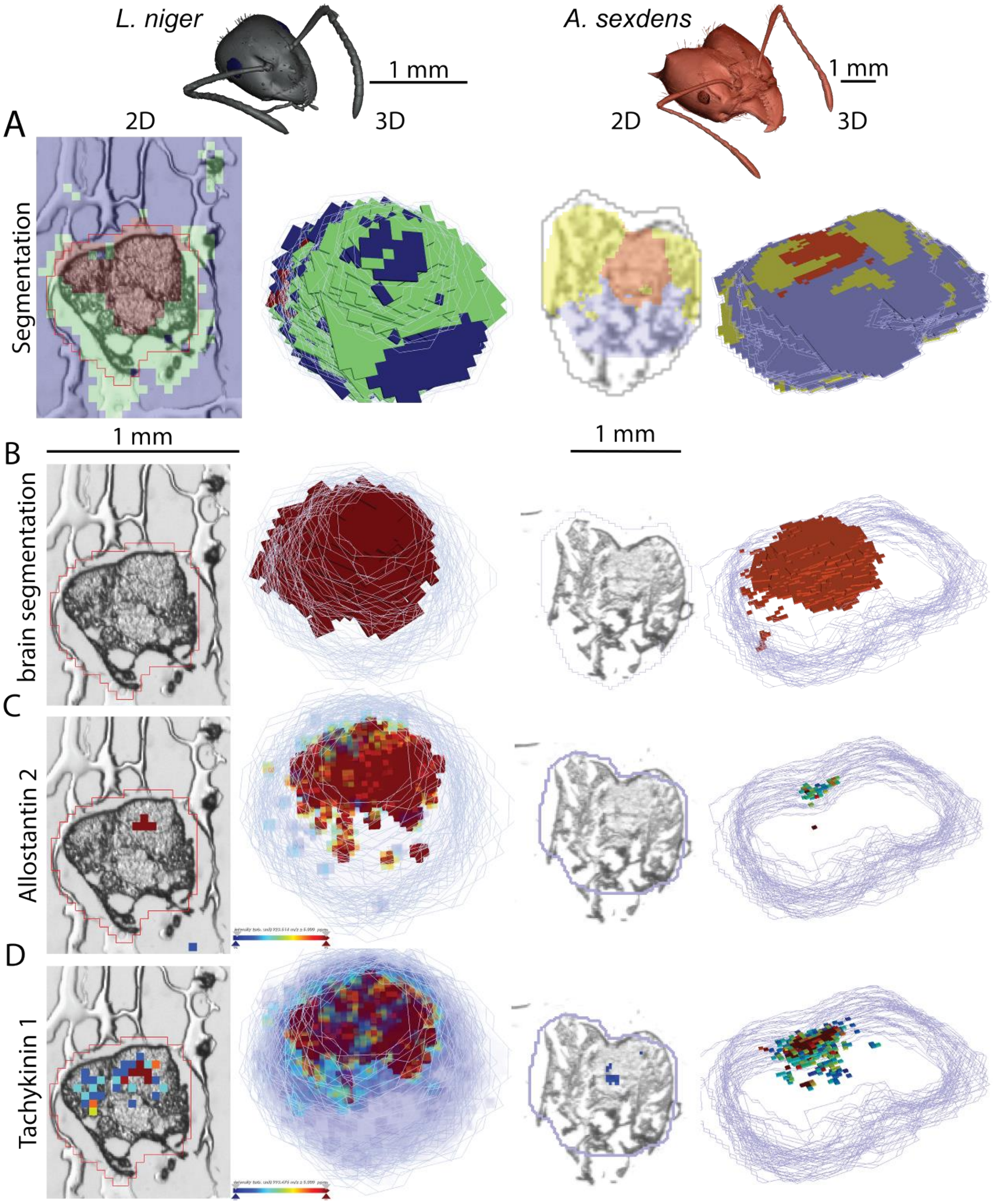

Supplementary Figure S2. Spatial clustering of MALDI-MSI data acquired from section series of *L. niger* and *A. sexdens* heads. (**A**) Dataset of each head divided into three spatial clusters of which one was distinctly colocalized with the brain (red) centered in the head capsule shown in (**B**). Tissues surrounding the brain of *L. niger* clustered within the green cluster and in *A. sexdens* summarized within a yellow and purple cluster. In *L. niger* off-tissue signals were assigned to the blue cluster. (**C**) and (**D**) show heat maps of the peptide distributions for allatostatin (AstA) 2 and tachykinin-related peptide 1 (TK1) for a 2D plane and the 3D volume for each ant, illustrating the spatial heterogeneity along depth.

Supplementary Figure S3. Penetration of the contrasting agent into the separated head capsules through the brain of *L. niger* (A-D) and *A. sexdens* (E-H). Magenta arrows indicate direction of diffusion. (**A, B, E, F**) show 2D planes and (**C,D,G,H)** show maximum intensity projections along the z-axis of the 3D volumes.

Supplementary Figure S4. Co-registration between mass spectrometry imaging and microtomography data. Workflow consisted of four main steps: 1. Matching the virtual plane in the µCT data to the physical sectioning plane of the tissue sections, 2. Allocate brain regions, visible in both modalities (µCT and bright-field microscopy sections used for MALDI-MSI), 3. Align MALDI-MSI data with corresponding µCT slice based on bright-field microscopy image in 3D space (using Amira software), 4. Create surface renderings of each neuropeptide measured with MALDI-MSI to colocalize and simultaneously visualize within 3D microanatomy of each animal.

Supplementary Table S1. Literature survey on MS imaging (MSI) of neuropeptides (most important references for this study)

Species	No. of peptides (by MSI) [remarks]	Year	Reference
primate (*Macaca mulatta*)	-[#]	2022	(1)
human	-	2022	(2)
crab (*Cancer borealis*)	7+11	2021	(3)
rat	-	2021	(4)
mollusc (*Aplysia californica*)	- [no MSI, Aplysia has giant neurons]	2021	(5)
snail (*Lymnaea stagnalis*)	-	2021	(6)
ant (*Cataglyphis nodus*)	16 [spatial organization obliterated, no clear anatomical context]	2021	(7)
crab (*Cancer borealis*)	-	2021	(8)
crab (*Cancer borealis*)	-	2020	(9)
mammalian	1	2020	(10)
crab (*Callinectes sapidus*)	-	2020	(11)
crab (*Cancer borealis*)	-	2020	(9)
cockroach (*Periplaneta americana*)	100 [from 15 precursor genes (including AstA, Crz, myosuppressin, NPLP1, sNPF), could be traced with an optimized protocol]	2019	(12)
rat	-	2019	(13)
honeybee (*Apis melifera*)[$]	-	2018	(14)
crustaceaen (*Callinectes sapidus* and *Carcinus maenas*)	-	2018	(15)
rat	-	2017	(16)
mammalian	-	2017	(17)
ant (*Cataglyphis fortis*)	-	2017	(18)
mammalian	-	2016	(19)
triclad (*Schmidtea mediterranea*)	-	2016	(20)
crab (*Carcinus maenas*)	-	2015	(21)
rat	-	2015	(22)
lobster (*Panulirus interruptus*)	51 / 7 [not in situ; unclear how many were detected by MSI]	2015	(23)
mammalian	4	2015	(24)
ant (*Camponotus floridanus*)	39 [indirect; dissection & LC/MS-MS of pooled ganglia]	2015	(25)
crab (*Cancer borealis*)	57 [single neuron resolution, but spatial information limited]	2014	(26)
honeybee (*Apis melifera*)	3	2014	(27)
human	7	2014	(28)
crab (*Callinectes sapidus*)	-	2013	(29)
crab (*Cancer borealis*)	12	2013	(30)
human	2	2013	(31)
crab (*Callinectes sapidus*)	-	2013	(32)
fly (*Drosophila melanogaster*)	-	2013	(33)
snail (*Helix pomatia*)	-	2012	(34)
rat	1	2012	(35)
crustacean	19	2012	(36)
mouse	-	2012	(37)
mollusc (*Aplysia californica*)	2	2012	(38)
rat	-	2011	(39)
mollusc (*Aplysia californica*)	- [neuronal cell culture]	2011	(40)
shrimp (*Penaeus monodon*)	-	2012	(41)
leech (*Hirudo medicinalis*)	-	2011	(42)
mouse	-	2010	(43)
cockroach (*Periplaneta americana*)	- [MS imaging on 30y-old (fixed & embedded) tissue sections; retrocerebral complex analysis – place of storage of abundant amounts of neuropeptides]	2010 & 2018	(44)
crab (*Cancer borealis*)	-	2010	(45)
lobster (*Homarus americanus*)	-	2010	(46)
mollusc (*Aplysia californica*)	-	2009	(47)
crab (*Cancer borealis*)	-	2009	(48)
rat	-	2008	(49)
beetle (*Tribolium castaneum*)	49(indirect)	2008	(50)
mollusc (*Aplysia californica*)	-(cells)	2007	(51)
crab (*Cancer borealis*)	30	2007	(52)
snail (*Lymnaea stagnalis*)	1	2005	(53)
cockroach (*Periplaneta americana*)	-(cells)	2005	(54)
fly (*Neobellieria bullata*)	-(indirect)	2004	(55)
mollusc (*Aplysia californica*)	-(cells)	2003	(56)

[*]Pubmed search terms (‘mass spectrometry AND imaging AND neuropeptides’ → 246 hits (of which are 24 reviews, and few methods description articles); ‘…AND tomography’ → no hits)
[#]-no. of peptides by MSI only unclear; used a combination of techniques (*in silico*, LC-MS/MS etc.)
[$]insects are highlighted in grey

Supplementary Table S2. A summary of *L. niger* and *A. cephalotes/A. sexdens* neuropeptide sequences, masses and MALDI MRMS results

Precursor		Peptide		*Lasius niger*			*Atta cephalotes / Atta sexdens*		
#	name	#	name	Peptide sequence (genome mining or sequencing)	HRM $[H]^+$ calc.	HRM $[H]^+$ exp. MALDI MRMS	Peptide sequence (genome mining (*A. cephalotes*) or sequencing (*A. sexdens*)	HRM $[H]^+$ calc.	HRM $[H]^+$ exp. MALDI MRMS
1	Adipokinetic hormone (AKH)	1	AKH	pQLNFSTGWGQ*	1119.513	n.f.	pQLNFSTGWGQ*	1119.513	n.f.
2	Allatotropin (AT)	2	AT	GFKPEYISTAIGF*	1428.752	n.f.	GFKPEYISTAIGF*	1428.752	n.f.
3	**Allatostatin A (AST A)**	3	AST A1	**LPLYNFGI***	935.535	n.f.	**LPLYTFGI***	922.540	n.f.
		4	AST A2	**TRPFSFGI***	923.510	923.513	**TRQFSFGI***	954.516	954.516
		5	AST A3	**LRDYRFGI***	1038.584	1038.584	**LRNYDFGI***	996.526	n.f.
		6	AST A4	**GGKPFSFGI***	908.499	n.f.	**GNHQFGFGI***	975.480	n.f.
		7	AST A5	**GWKLATGETAVS***	1218.648	n.f.	**VWKLATGETAVS***	1260.695	n.f.
4	Allatostatin C (AST C)	8	AST C1	LRSQLDIGDLQR	1413.781	n.f.	HFRNQMNVNDLQR	1671.813	n.f.
		9	AST C2	SYWKQCAFNAVSCF*	1650.719	n.f.	NFWRQCSLNAVACF*	1655.757	n.f.
5	Allatostatin CC (AST CC)	10	AST CC	GQPKGQVYWRCYFNAVTCF	2265.037	n.f.	ALAQSELFWRCYFNALACF	2251.046	n.f.
6	CAPA	11	CAPA1	SAGLVPYPRI*	1071.631	1071.634	SAGLVAYPRI*	1045.615	n.f.
		12	CAPA2	ALGIIHQPRI*	1116.700	1116.704	AFGIIHKPRIG*	1207.742	n.f.
		13	CAPA3	NTQGQGGYTPRL*	1290.655	n.f.	NSQGQGGYTPRL*	1276.639	n.f.
7	Crustacean cardioactive peptide (CCAP)	14	CCAP	PFCNAFTGC*	956.37535	n.f.	PFCNAFTGC*	956.37535	956.36688
8	CCHa2	15	CCHa2	GCASFGHSCFGGH*	1263.478	n.f.	GCAAFGHTCYGGY*	1303.498	n.f.
9	CCHa1	16	CCHa1	SGGCASFGHSCFGGH	1407.531	n.f.	AETCLHFGNSCWAH*	1572.647	n.f.
10	Corazonin (CRZ)	17	CRZ	pQTFQYSRGWTN*	1369.629	n.f.	pQTFQYSRGWTN*	1369.629	1369.631
11	Diuretic hormone (DH44/CRF-like)	18	DH44/ CRF-like	IGSLSIVNNLDVLRQRVLLELARRKAQQDQRQIDANRRVLESI*	4995.851	out of range	IGSLSIVNNLDVLRQRVLLELARRKALQDQRQVEENRRFLESV*	5072.867	out of range
12	Diuretic hormone (DH31-like)	19	DH31-like	GLDLGLNRGFSGSQAAKHLMGLAAANYAGGP*	3013.542	out of range	GLDLGLNRGYSGSQAAKHMMGLAAANYAGGP*	3047.494	out of range
13	Ecdysis triggering hormone (ETH)	20	ETH1	DEVPAFFLKIAKIPTLPRV*	2153.284	2153.286	EEVPAFFLKIAKIPTLPRV*	2167.300	n.f.
		21	ETH2	SGRFEDFFYKAEKHIPRI*	2239.177	n.f.	SGRFEDFFYKAEKHIPRI*	2239.177	n.f.
14	FMRFamide-like peptides (FMRFa)	22	FMRFa 1	STMGSSFIRF*	1131.562	n.f.	STMGSSFIRF*	1131.562	n.f.
		23	FMRFa 2	WKSPDVVIRF*	1245.710	n.f.	WKSPDIVIRF*	1259.726	n.f.
		24	FMRFa 3	GKNDLNFIRF*	1222.669	n.f.	GRNDLNFIRF*	1250.675	n.f.
15	IDLSRF-like	25	IDLSRF-like	IDLSRFYGHINT	1435.733	1435.746	IDLSRFYGHFNT	1469.717	1469.720
16	Inotocin	26	Inotocin	CLITNCPRG*	973.471	n.f.	CLITNCPRG*	973.471	n.f.
17	Insulin-like peptide B (ILP-B)	27	ILP-B	PTRNVPQKYCGKKLSNALQIICDGVYNSMF	3385.685	out of range	SAVSAPQKYCGKKLSNALQIICDGVYNSMF	3233.579	out of range
18	Insulin-like peptide (ILP-C)	28	ILP-C	QVTLRKSHIRMQKLCSRSLSDALYLVCKERGYNEPFSYSGEDEPRGDSGPGLVEECCYHSCSYEQLERYCKPLPEE	8791.098	out of range	DNVIFKKSHQRMQKLCSRKLSDALQVMCRDRGYNEPFYSNEDESRIDPGPGLVEECCYHQCTYEQMEQYCKPLPAE	8976.106	out of range
19	ITG-like	29	ITG-like	ITGQGNRLF	1005.548	1005.551	ITGQGNRLF	1005.548	1005.548
20	**Myosuppressin (MS)**	30	MS	**pQDVDHVFLRF***	1257.638	1257.642	**pQDVDHVFLRF***	1257.638	1257.639
21	Neuropeptide F (NPF)	31	NPF	ESMARSTRPKAIVNSEELKRYADLVNDFYYLTTKPRY*	4424.282	out of range	EPEPMTRLTRPPQITNSNVQLQRYADSVKNFYHMYGKSRH*	4788.400	out of range
22	Neuropeptide-like precursor 1 (NPLP1-1)	32	NPLP1 1	SLATLAKNDDLPITLQMREGNSDDEE	2875.363	out of range	SLATLAKNDDLPFIVQEREDNGDDEE	2933.365	out of range
		33	NPLP1 2	NVGTLARDFALPT*	1373.754	1373.751	NVGALARDFALPT*	1343.743	n.f.

		34	NPLP13	HIASVARDHGLPN*	1385.740	1385.746	HIGSVLRDYSTMS*	1464.726	n.f.
		35	NPLP14	NIGSLARQSTLPSN*	1456.787	1456.800	NIGSLARQSMLPIS*	1485.821	1485.822
		36	NPLP15	NVASLARYYMLPQN*	1638.842	n.f.	NVASLARDSMLPQN	1515.758	n.f.
		37	NPLP16	NVAALARDSSLPY*	1375.733	1375.743	NVAALARDSSLPY*	1375.733	1375.735
		38	NPLP17	YLGSLARNGGYIPVREYDED	2287.099	n.f.	YLGALVRNGGYPIRGYDE*	2012.035	n.f.
		39	NPLP18	SIASLARNADWPSVV	1585.833	n.f.	NIASLARNADWPGFM	1662.806	n.f
23	Orcokinin (OK)	40	OK1	NFDEIDRSVFDRFS	1746.808	n.f.	NFDEIDRSVFDHFS	1727.766	n.f.
		41	OK2	NIDEIDTAFDSFF	1533.674	n.f.	NIDEIDTAFDSLF	1499.690	n.f.
		42	OK3	NFDEIDRVGWGGFV	1610.760	n.f.	NFDEIDRAGWGGFV	1582.728	1582.732
		43	OK4	LNNYLADRRQQ or LNNYLADRRQ	1390.719 or 1262.660	n.f.	LNNYLADRRQQ or LNNYLADRRQ	1390.719 or 1262.660	n.f.
24	Pigment-dispersing-factor (PDF)	44	PDF	NSELINSLLSLPKNMNNA*	1971.033	n.f.	NSELINSLLGLPKNMHNA*	1964.038	n.f.
25	Pyrokinin (PK)	45	PK1	TTAQEITSGMWFGPRL*	1793.900	n.f.	TTQDITSGMWFGPRL*	1708.848	1708.852
		46	PK2	pQPTQFTPRL*	1069.570	n.f.	pQPSQFTPRL*	1055.555	n.f
		47	PK3	GSEEELFSYGDETDRNEIDEDDHVLPPIFAPRL*	3803.767	out of range	GSSEDLSSYGDSYEIDEDDRLLPPIFTSRL*	3376.570	out of range
		48	PK4	VPWIPSPRL*	1063.641	n.f.	FPWIPSPRL*	1111.641	n.f.
26	**Short neuropeptide F (sNPF)**	49	sNPF	**SPSLRLRF***	974.589	974.592	**SPSLRLRF***	974.589	974.589
27	SIFamide	50	SIFamide	GYKKPPFNGSIF*	1353.731	n.f.	AYKKPPFNGSIF*	1367.747	n.f.
28	sulfakinin-like peptide (SK)	51	SK	QQLDDYGHMRF*	1408.643	n.f.	QQLDDYGHMRF*	1408.643	n.f.
29	**Tachykinin-related peptide (TK)**	52	TK1	**APMGFQGMR***	993.476	993.4873	**APMGFQGMR***	993.476	993.476
		53	TK2	n.f.			**APKGFQGMR***	990.530	990.531
		54	TK3	**LLAMGFQGIR***	1104.635	n.f.	**ASMGFQGMR***	983.455	983.456
		55	TK4	**TVMGFQGMR***	1025.502	1025.504	**TLMGFQGMR***	1039.518	1039.518
		56	TK5	**SPFRYFEMR***	1231.604	n.f.	n.f.		
		57	TK6	**NPRWELRGMFVGVR***	1715.927	n.f.	n.f.		
		58	TK7	**AAMGFYGTR***	972.472	972.472	**AALGFYGTR***	954.516	954.516
30	True sulfakinin (SK)	59	tSK	SNGNDEEYGHSRF*^{s}	1590.5878	n.f.	FNGDDEEYGHSRL *^{s}	1617.6238^{s}	n.f.

***** – amide group; **p** – pyro group; **n.f.** – not found; ^{s} – *O*-sulfated tyrosine; **HRM** – high resolution mass; **calc.** – calculated.
Cysteines shown in red (C) form disulphide bond
Peptides highlighted in red were found in both species
Precursors and peptides shown in **bold** were confirmed by sequencing of the precursor genes
Masses in highlighted blue were detected with mass error less than 5 ppm to theoretical mass
Masses in highlighted green were detected with mass error between 5 and 10 ppm to theoretical mass

Supplementary Table S3. Resolved identified brain areas in *L. niger* and *A. sexdens* by using µCT

Brain, subesophageal ganglion and nerves	*Lasius niger*	*Atta sexdens*
**Brain regions and SEG**		
OL optic lobe	+	+
ME medulla	+	+
LO lobula	+	+
LA Lamina	+	-
MB mushroom body	+	+
VL vertical lobe	+	+
ML medial lobe	+	+
CA calyx	+	+
m-CA medial calyx	+	+
l-CA lateral calyx	+	+
PE pedunculus	+	+
CX central complex	+	+
CB central body	+	+
CBU central body upper unit	+	-
CBL central body lower unit	+	-
PB protocerebral bridge	+	-
NO noduli	+	-
SP Superior protocerebrum	+	+
IP inferior protocerebrum	+	+
VPLNP ventro-posterolateral neuropils	+	+
VMC ventromedial cerebrum	+	+
AL antennal lobe	+	+
LAL lateral accessory lobe	+	+
AMMC antennal mechanosensory and motor centre	+	+
SEG subesophageal ganglion	+	+
**Nerves**		
Optic nerve	+	+
Antennal nerve	+	+
Mandibular nerve	+	+
Maxillary nerve	+	+
Labial nerve	-	-

Supplementary Table S4. Literature review on the biological function/localization of ant (insect) neuropeptides (relevant for this study)

Peptide family	Biological function/localization	Species	Reference
Allatotropin (AT)	see Allatostatin A		
Allatostatin A (AST A1-A5)	- potential modulator of behavioral transitions: expression is significantly increased in foragers compared to interior workers<br>- widespread localization: AstA-ir neurons innervate primary sensory neuropils and higher order integration areas [AstA-ir was observed in the fan-shaped body of the central complex, antennal lobe (dorsolateral and dorsomedial cell clusters), optic lobe/lamina-medulla-lobula, superior medial protocerebrum, extrinsic mushroom body neurons of the vertical lobe projecting to the calyces; AT-ir was more restricted]<br>- immunolocalized in central body, collar of the mushroom bodies' calyces, antennal lobes/antennal glomeruli, central adjoining neuropils, vertical lobe, protocerebral-calycal tract, pedunculus, optic lobes/lobula-medulla-lamina, superior medial protocerebrum, lateral accessory lobe, ventrolateral neuropils, lateral horn, inferior neuropils, peri-oesophageal neuropils, etc.	*Cataglyphis nodus*	(57)
	2D brain maps (single sections) for AstA, CRZ, IDL, ITG, MS, NPLP1, sNPF, TK, etc.		(7)
	MS and immunolocalization: AstA and allatotropin (AT) were suggested as potential candidate regulators of behavioral changes in the age-related polyethism; MS showed presence of these peptides in central brain and ventral ganglia; immunolocalization showed that AstA is present in primary sensory processing centers and in higher order integration centers	*Cataglyphis fortis*	(18)
	see Tachykinin-related peptide		
Corazonin (CRZ)	- social behavior (caste identity); well conserved peptide (also the localization of the CRZ-producing neuronal cell bodies!)	*Harpegnathos saltator*	(58)
	- 2D brain maps (single sections) for AstA, CRZ, IDL, ITG, MS, NPLP1, sNPF, TK, etc.<br>- produced in a cluster of four (CRZ-ir) neuronal cell bodies in the lateral part of each brain hemisphere (*pars lateralis*), sending axons towards the medial protocerebrum (superior medial protocerebrum, flange; a bundle runs in between the lateral and median mushroom body calyces towards posterior brain areas) and the (neurohaemal) retrocerebral complex (incl. the storage part of the *Corpora cardiaca*)<br>- CRZ gene expression as well as the volume of the CRZ-expressing neuronal cell bodies are significantly increased in foragers compared to interior workers	*Cataglyphis nodus*	(7, 57)
IDLSRF-like	2D brain maps (single sections) for AstA, CRZ, IDL, ITG, MS, NPLP1, sNPF, TK, etc.	*Cataglyphis nodus*	(7)
Inotocin (INT)	regulation of genes involved in metabolism: knock-down ants exhibited higher walking activity and increased self-grooming in the brood chamber →INT signaling may be important for regulating metabolic processes and locomotion locomotion	*Lasius neglectus*	(59)
ITG-like	2D brain maps (single sections) for AstA, CRZ, IDL, ITG, MS, NPLP1, sNPF, TK, etc.	*Cataglyphis nodus*	(7)
Myosuppressin (MS)	2D brain maps (single sections) for AstA, CRZ, IDL, ITG, MS, NPLP1, sNPF, TK, etc.	*Cataglyphis nodus*	(7)
Neuropeptide-like precursor 1 (NPLP1 1-8)	2D brain maps (single sections) for AstA, CRZ, IDL, ITG, MS, NPLP1, sNPF, TK, etc.	*Cataglyphis nodus*	(7)
	NPLP1 derived peptides were observed in a peptidomics analysis of CNS; the status of NLP1 as a 'bona fide' neuropeptide precursor was/is not beyond any doubt (therefore designated as "neuropeptide-like"), but peptides derived from this precursor are frequently observed in mass spec analyses of insect brains; a peptide (NLP1-VQQ), which is derived from this precursor, can act as a ligand for the receptor guanylate cyclase Gyc76C; this receptor is most highly expressed in immune and stress-sensing epithelia, such as the malpighian tubules (MT), regulates organismal survival, and modulates the IMD immune pathway in response to salt stress. NPLP1-VQQ increases the cGMP content and fluid secretion in the MT.	*Drosophila melanogaster* (fruit fly)	(60)
	Functional information regarding the role of (other) NPLP1 derived peptides is scarce; a significant reduction of the levels of two NPLP1-derived peptides in the CNS 24 hrs after blood feeding, while one of these peptides was first significantly upregulated 4 hrs after blood feeding (before being reduced after 24hrs).	*Rhodnius prolixus* (bloodsucking bug)	(61)
Short neuropeptide F (SNPF)	2D brain maps (single sections) for AstA, CRZ, IDL, ITG, MS, NPLP1, sNPF, TK, etc.	*Cataglyphis nodus*	(7)
	MS and immunolocalization: sNPF was suggested as potential candidate regulator of behavioral changes in the age-related polyethism; MS showed presence of these peptides in central brain and ventral ganglia; sNPF was the only one that could be detected in the retrocerebral complex	*Cataglyphis fortis*	(18)
	An sNPF receptor has been identified and immunolocalized: the immunoreactivity appeared to be mainly distributed in or near important sensory (processing/integrative) neuropils, such as mushroom bodies, antennal lobes, central complex, different regions of the protocerebrum and suboesophageal ganglion, and based on this it was suggested to be linked with circuits regulating learning and feeding behaviors (the receptor-ir was also detected in the ovary of mated queens)	*Solenopsis invicta*	(62)

	investigated whether sNPF would be capable of modulating the acquisition (learning) and formation of appetitive visual (colour) memories → sNPF plays a role in motivational processes of foraging, as well as in cognitive processes associated with it	*Apis melifera* (honeybee)	(63)
sulfakinin-like peptide (SK)	see Tachykinin-related peptide		
Tachykinin-related peptide (TK1-7)	- 2D brain maps (single sections) for AstA, CRZ, IDL, ITG, MS, NPLP1, sNPF, TK, etc.<br>- spatial distribution of 22 peptides derived from 16 precursors; obtained from consecutive brain sections of the ant were compared with TK-ir and showed a 'relatively good' match (note that the authors also mentioned some 'slight' differences); the authors of this paper proposed neuropeptides as 'powerful' candidates for modulating the age-related behavioral transitions occurring in ants (the data shown for TK seem to be more detailed)	*Cataglyphis nodus*	(7, 57)
	- MS and immunolocalization: TK peptides were suggested as potential candidate regulators of behavioral changes in the age-related polyethism; MS showed presence of these peptides in central brain and ventral ganglia; immunolocalization showed that TK peptides are present in primary sensory processing centers and in higher order integration centers [TK-ir was observed in extrinsic mushroom body neurons projecting from the vertical lobe projecting to dorsal brain parts, the antennal lobe (lateral cell clusters), the fan-shaped body of the central complex, the (visual) collar region (not in the olfactory lip) of the calyx, optic lobe/ medulla-lobula (not in the lamina)]<br>- TK-ir changed markedly in the central complex 1, 7 and 14 days post-eclosion [D1: TK-ir clearly visible in layers I and II of the fan-shaped body; D7: weak TK-ir, but TK-ir appears in the ellipsoid body; D14: TK-ir restricted to layer II of the fan-shaped body of the central complex], and these age-associated changes may be related to locomotion behavior	*Cataglyphis fortis*	(18)
	- widespread localization: TK-ir neurons innervate primary sensory neuropils and higher order integration areas; immunolocalized in the ant (central body, pedunculus, vertical lobe, antennal lobes/antennal glomeruli, antennal mechanosensory and motor center and prow, all superior (lateral, intermediate, medial) neuropils, central adjoining neuropils, protocerebral-calycal tract, optic lobes/lobula-medulla, peri-oesophageal neuropils, lateral accessory lobe, ventrolateral neuropils, lateral horn, inferior neuropils, ventromedial neuropils, etc.)<br>- TK gene expression did not differ between foragers and interior workers	*Cataglyphis nodus*	(57)
	response thresholds to task-specific stimuli corresponding to the specialization of three behavioral phenotypes; quantitative comparison of neuropeptidome: two <u>TK related peptides (TRP2 and TRP3)</u> as candidate modulators of the response thresholds; peptide injections and RNAi knockdown consistently affected responsiveness to task-specific stimuli for each specialized phenotype in opposite ways; TK (=TRP) signaling regulates task-specific responsiveness in behaviorally specialized workers; and they suggested that TK signaling may generally control the context specificity of animal behaviors]	*Apis melifera* and *Apis cerana* (honeybee)	(64)
	quantitative (by differential isotope labeling); robust and dynamic regulation in function of nectar/pollen collection; peptides include: **<u>TK</u>** and sNPF, which showed the strongest changes in association with nectar and/or pollen foraging (note: that the experiment also analyzed changes in peptide abundances before and after the food collection; IDLSRF-like peptide levels were changing); several peptides that are involved in regulating food intake in solitary insect species may have evolved (in social insects such as the honey bee) towards a ('more specialized') role in social foraging	*Apis melifera* (honeybee)	(65)
	created a high-resolution atlas of the central complex; this brain region is conserved among insect species and consists of neuropils that underpin behaviors, such as goal-oriented locomotion (incl. "sky-compass" orientation); to structurally describe this brain region in detail, immunolocalizations of different neuropeptides were performed, i.e. **<u>TK</u>**, FMRFa, SK and AstA	*Apis melifera* (honeybee)	(66)

Notes:
- changes (or local differences) in peptide abundance may be based on differences in synthesis (peptide precursor gene expression, transcript splicing, precursor processing, turnover rate, etc.), storage/release, diffusion/transport, stability/degradation, …
- intensity in MS may also depend on ionization/detection efficiency
- neuropeptides are usually multifunctional (pleiotropic); within the CNS they may act in structurally and functionally (partially or completely) distinct neuronal circuits (with different regulation and roles)

Supplementary Table S5. DNA oligonucleotides used in for PCR and sequencing of neuropeptide precursors

Precursor name	Oligonucleotide name	Oligonucleotide sequence	PCR product, bp
A. sexdens tachykinin-related peptide	A.sex-TK-Fw	AGAATGCTTTTTAGTTCGGTTC	716
	A.sex-TK-Rev	ATGCCCTGAAAACCCATAAGG	
	3'-RACE-RT-primer	GGCCACGCGTCGACTAGTACTTTTTTTTTTTTTTTTT	361
	3'-RACE-PCR-Rev	CTACTACTACTAGGCCACGCGTCGACTAGTAC	
	3'-RACE-PCR-Fw	AGGGCTTCTATGGGATTTCAGG	
A. sexdens short neuropeptide F	A.sex-sNPF-Fw	ATGTACGCCAAATATTACGCAG	333
	A.sex-sNPF-Rev	TTAATTGTTGTCGTCATATCCC	
A. sexdens allatostatin	A.sex-Ast-Fw	ATGAAGACAGCAACAAGTTTAATT	583
	A.sex-Ast-Rev	TTACTGATTCAAATCCTCATTCTCA	
A. sexdens myosuppressin	A.sex-Myo-Fw	ATGATGAGCTCGACGCTGATG	293
	A.sex-Myo-Rev	TGGTTCTAACGTCGTTTTCC	
L. niger tachykinin-related peptide	L.nig-TK-Fw1	AAACTCGAAACGGATCGTCGAA	798
	L.nig-TK-Rev1	CCATGACGGTTCGTTTCTCAAG	
	L.nig-TK-Fw2	CTTCTCGCGATGGGATTTCAAG	773
	L.nig-TK-Rev2	CGACAGCATTTGCATCCATCAT	
L. niger allatostatin	L.nig-Ast-Fw	GCGAAATCGCGCTGATAGTTTC	678
	L.nig-Ast-Rev	GGAGGAGAACAATCCGTGCTTC	
L. niger short neuropeptide F	L.nig-sNPF-Fw	CTCTCTCTCATCCCCCATCGAA	474
	L.nig-sNPF-Rev	AATTGGAAAGGTGTCCCGATCA	
L. niger myosuppressin	L.nig-Myo-Fw	CGATACATGCACATGCACATCC	471
	L.nig-Myo-Rev	GCGTACGTGCGGTGACTGTAAA	

Supplementary Data S1. Sequenced full or partial neuropeptides precursors (myosuppressin, tachykinin-related peptide, allatostatin and short neuropeptide F) of *A. sexdens or L. niger*

Only sequences (sometimes partial), which were confirmed by sequencing are shown; *indicates a STOP codon; dots indicate partial sequences (missing N- or C-termini); in positions marked in yellow two different nucleotides were determined during sequencing.

>Atta sexdens myosuppressin precursor
ATGATGAGCTCGACGCTGATGATCCTCGTATCCGTGACGACTATGGCAGTCCTTTCCGGCGAAGtTCTCGCCGGATCGCCTGG
TATTTCATGCAAcCCAGGATTTCTTGATGAACTTCCACCCAGATTCCGTAAAATCTGCATGGCATTCGCCAGAATATGGGATG
TTAGGGACATGAATGACTTCATTGACGACAAAGAATACCGAGAGAACTTACCGCGGTACGAtAGCACTGTCAAGAGACAAGAT
GTCGATCATGTTTTCTTGCGCTTTGGAAAACGACGTTAGAACCA
T or C (ambiguous), no change in a.a. sequence

>Atta sexdens myosuppressin precursor
MMSSTLMILVSVTTMAVLSGEVLAGSPGISCNPGFLDELPPRFRKICMAFARIWDVRDMNDFIDDKEYRENLPRYDSTVKRQD
VDHVFLRFGKRR*

>Atta sexdens tachykinin-related peptide precursor
TCGGTTCTTTTCCTGGCAGTCTGGACTAGTTCGTCATTCGCAGAGGAATCCTCCAATGATGCCGCGTCTGCCAAACGAGCGCC
CATGGGATTTCAAGGTATGCGCGGAAAAAAAGACCTCATTCCTACGGTCGCAGAACACAATGAACTTTCCAAAAGAACGTTAG
TGAATTTTCAGGATAAGGATTCGAGCGCGTCCGAAATTGAGGACAATCTTCTGCACGATGAGTTTGACAAAAGAGCACCGAAG
GGATTTCAGGGTATGAGGGGGAAAAAGGATTATTTGATACCTGATTTTGAAGACTCCTACTTTCTTGAGGACTACGACAAAAG
AGCGCCAATGGGTTTTCAGGGTATGAGAGGCAAGAAGGCTATATTAGAAGACGAATATTATAAACGTGCACCTATGGGATTTC
AAGGAATGAGAGGGAAGAAATCTTTGGAGGAGGTGCTGAGTGAAATTGAAAAGAGAGCTGCGTTGGGCTTTTACGGTACTAGA
GGAAAAAAAACATATATTTTCGAGTATCCACAAGATTACGAAAAGAGACTTTTAGCAATGGAATTCCAAGATATGCACAATAA
GATAAAAGAAGAATGGGAAAAAAGGGCTTCTATGGGATTTCAGGGGATGAGAGGCAAGAAAGCATTGTATGACGAAATAGAAG
AACTTGAGAAAAGAACCCTTATGGGTTTTCAGGGCATGAGAGGCAAGAAAGACGGCTTTGAAAATTACATAGATTATTACATA
GATGATCCCGACATGGATTTTGACAAGAAGGCATCAATGAGTTTTCAAGGGATGAGAGGCAAGAAAGACACCGATAAGAGAGC
ACCGATGGGTTTTCAAGGTATGCGGGGCAAGAGGAGTGTAGCACAAAGATTTGAGCCCAGCATGAATTTTGGACCATTAAACG
AATACCAAGATTCACAATAATACAACAACTCTTAGAAACGATAATGCAACTGTTACGAAACGTCTCCTCCAAATAATTAAACC
GATATAGCTAAATCTAAAAAAAAAAAAAAAAAAAGTTTT
A or C (ambiguous), D (aspartic acid) or A (alanine) in a.a. sequence

> Atta sexdens tachykinin-related peptide precursor
...SVLFLAVWTSSSFAEESSNDAASAKRAPMGFQGMRGKKDLIPTVAEHNELSKRTLVNFQDKDSSASEIEDNLLHDEFDKR
APKGFQGMRGKKDYLIPDFEDSYFLEDYDKRAPMGFQGMRGKKAILEDEYYKRAPMGFQGMRGKKSLEEVLSEIEKRAALGFY
GTRGKKTYIFEYPQDYEKRLLAMEFQDMHNKIKEEWEKRASMGFQGMRGKKALYDEIEELEKRTLMGFQGMRGKKDGFENYID
YYIDDPDMDFDKKASMSFQGMRGKKDTDKRAPMGFQGMRGKRSVAQRFEPSMNFGPLNEYQDSQ*
D or A in a.a. sequence

>Atta sexdens allatostatin precursor
AACAAGTTTAATTACTATACGAATTATTATGTTCTATCTGTTGAGCGTTGTTGGACGATCAGCAGTGGCAATAGAAGAAGCAT
CTTCCTCGTCTTTACACATTCAACGATTGCATCCGTTATTAAACAACATGGAATATAATAAAGAACCTATGAAAAAGGCATAC
ATTGCCGAATATAAGAGATTACCCCTTTATACCTTCGGTATTGGAAAACGATGGATTGACAATAATGAAGATAAACGAACCCG
GCAATTCTCGTTCGGTATTGGCAAGCGTCTCCGAAATTACGATTTCGGTATAGGAAAGCGCAACAATGAATATCATCCTTTGA
GTTTGGACTATTTCTTGGTCGATAACATGGGGAACTATCAATCTCACGAGGACAACTCAAATGATTTTATAGAAAACAAACGT
GGTAACCATCAATTCGGCTTTGGAATTGGAAAACGAGTTTGGAAATTGGCGACTGGAGAAACGGCTGTATCCGGAAGAAGATT
AAACGATGTTATAGTCCCGAAATACTGGTTCAGTACTTTGACCAAAGAACTAGATGAGAATGAGGATT

> Atta sexdens allatostatin precursor
...TSLITIRIIMFYLLSVVGRSAVAIEEASSSSLHIQRLHPLLNNMEYNKEPMKKAYIAEYKRLPLYTFGIGKRWIDNNEDK
RTRQFSFGIGKRLRNYDFGIGKRNNEYHPLSLDYFLVDNMGNYQSHEDNSNDFIENKRGNHQFGFGIGKRVWKLATGETAVSG
RRLNDVIVPKYWFSTLTKELDENED...

>Atta sexdens short neuropeptide F (sNPF) precursor
AATATTACGCAGCTTTCATTCTTGTCGTCGTGATAGTCGGTCTCGTGGACGCTACCGAAAATTACATGGATTACGGAGAAGAA
ATGGCGGAAAAAGCACCTGCAGAGAATATCCATGAGCTGTATAAGCTCTTGCTACAACGCAATGCGCTCGACAACGTCGGCTT
TGGCGGCATCCCGTTGGAACACCTGATGATTCGAAAGTCGCAGCGATCGCCATCGCTGCGTCTTCGATTTGGTCGTTCTGGGC
AACACATCTCCGCGGGAGCACTGCCGAGACCCTTAGGCGCGGCGGCTGCGGCGGGATATGACGA

>Atta sexdens short neuropeptide F (sNPF) precursor
…YYAAFILVVVIVGLVDATENYMDYGEEMAEKAPAENIHELYKLLLQRNALDNVGFGGIPLEHLMIRKSQRSPSLRLRFGRSG
QHISAGALPRPLGAAAAAGYD...

>Lasius niger myosuppressin precursor
AAAATGATGAGCTCGACATTGATGATTCTCGTATCTATGACGACCATGGCACTTTTGTCCGGCGAAGCTTTCGCCGCGTTGCC
CGCTCAATGTAATTCAGGTTTTCTCGAGGAACTTCCACCTAGACTGCGTAAAATCTGTATCGCTATCGCCAGAATATGGGATG
CCAGGGAAATGAACGATTTCGTCGACGACAGAGAATATCGAGAGAACTTGCCCCGTTATGACAGCAGTGTCAAGAGGCAAGAC
GTCGATCATGTTTTCTTGCGCTTCGGGAAACGACGTTAAAAACAACGGCCTGCTCTGTATTCGCACTAGAGATAAACGATCTC
AAGTTCTCTTTCAATTATTCGATCGCCCCGTCATCGATTCGTAGGACATTTGCCGAATGTAAAAAAAAAAAAA

>Lasius niger myosuppressin precursor
MMSSTLMILVSMTTMALLSGEAFAALPAQCNSGFLEELPPRLRKICIAIARIWDAREMNDFVDDREYRENLPRYDSSVKRQDV
DHVFLRFGKRR*

>Lasius niger tachykinin-related peptide precursor
TTCTTATCGATTTCACTGTTACGTCGCCATATTTTTTGACACTTTCAGTCTTCCTTTTGAAAGAATGCTTATCAACTCAGTTC
TTTTTCTGGCAGTCTGGATCAGTTCGTCGCTTGCGGAAGAATTATCTCCCAACGATGCTACGTCCGCCAAACGGGCGCCTATG
GGATTTCAAGGTATGCGCGGGAAGAAAAATCTCATCCCCACAGAACACAATAAATTGTCCAAGAGAACGCTAATGGATTTTCA
AGATACGCGCGATAATAAGGATTCGAATGCGCCTGACATTGAAGATAATCTTTTGCACGACGAGTTTGACAAGCGGGCTCCAA
TGGGATTTCAGGGTATGAGAGGGAAGAAAGATTATTTGATGCCTGATTTCGAGGATTCCTACTTTCGTGACGAAAAAAGAGCA
CCAATGGGTTTTCAGGGTATGAGAGGCAAGAAAATAGTTTCGGATGATGAATATTATAAACGAGCACCGATGGGATTTCAAGG
AATGAGAGGAAAGAAGTCTTCGGAAGAAGTGTTAGATGAAATTGAAAAGAAGGCCGCGATGGGCTTTTATGGTACGAGGGGAA
AAAAGACATACGTTCTCGAGTATCCGGAAGATTACGAAAAGAGACTTCTCGCGATGGGATTTCAAGGTATTCGCGGCAAGTTG
AAAGAATTTCCGGTAGAGTGGGAGAAAAGGGCTCCCATGGGATTTCAGGGGATGAGAGGCAAGAAAGCATTGCTTGACGAGAT
AGAGGAACTTGAGAAACGAACCGTCATGGGTTTTCAGGGCATGAGAGGCAAGAAAAATGCCTTTGAAAATTACGTGGATTATT
ACATGGATCCCGACATGGATTTCGACAAGAGAGCACCAATGGGTTTTCAAGGAATGAGAGGCAAAAAAGATTCCGATAAAAGG
GCACCTATGGGTTTTCAAGGCATGAGAGGCAAGAGAAATACGGGACAAAGATTTGATACTGGCATGGACTTTGAAACTCGAAC
ATCAAACGAATATCAAGGAACGAGCAATAGAAGAAACGCTCTAGCCTCGTGCCAGCTCGAAAAACGATCGCCCTTCCGATATT
TCGAGATGCGCGGTAAGAAAAATCCACGATGGGAATTACGGGGGATGTTTGTGGGGGTAAGAGGCAAAAAATGGGCGACAGCC
CCGTACGAGGACGACAGCCCGTTCATAAGCGTGTTTGATAACACCGAAAGGATCGGCGTGGATGGAGATTCGCCAGCAATATT
AGATTCACAATAATGCAGCAGCCCTTAGAAACGATAACTGTTACGAAACATCTTCTCCAAATACATAATTAAACCGAGCTAAA
CCTAAATAGCTAAATCTAAAAG

>Lasius niger tachykinin-related peptide precursor
MLINSVLFLAVWISSSLAEELSPNDATSAKRAPMGFQGMRGKKNLIPTEHNKLSKRTLMDFQDTRDNKDSNAPDIEDNLLHDE
FDKRAPMGFQGMRGKKDYLMPDFEDSYFRDEKRAPMGFQGMRGKKIVSDDEYYKRAPMGFQGMRGKKSSEEVLDEIEKKAAMG
FYGTRGKKTYVLEYPEDYEKRLLAMGFQGIRGKLKEFPVEWEKRAPMGFQGMRGKKALLDEIEELEKRTVMGFQGMRGKKNAF
ENYVDYYMDPDMDFDKRAPMGFQGMRGKKDSDKRAPMGFQGMRGKRNTGQRFDTGMDFETRTSNEYQGTSNRRNALASCQLEK
RSPFRYFEMRGKKNPRWELRGMFVGVRGKKWATAPYEDDSPFISVFDNTERIGVDGDSPAILDSQ*

>Lasius niger allatostatin precursor
CGCGCTGATAGTTTCGCAGCAACAGATAGATCTGAACCAGCATCATGAAGTCAAAAACGAGTCTGATCGCTATGAGAATCATT
ATGTTCTACCTGTTGAGCGTCGTTGGACGATCAACAGCGGCAATGGAAGAGGCACCACTCTCGTCTTTGCATATTCCACGATT
GAATCCGTTATCGAGCAACTTGGAGTACGACGAACCCTCTGAAAAAAGAGCGTACGCTTACATTTCCGAATACAAGAGGCTAC
CTCTTTACAACTTCGGTATTGGAAAGCGATGGATCGATAATAGCGATGATAAACGGACGCGGCCGTTCTCGTTCGGCATCGGA
AAACGTCTCAGGGACTACAGGTTCGGCATAGGAAAGCGTAATAGCGGATACCGTCCCTTGGGCATGGATTATTTCTCGGTCGA
CAACATGGAGGGCTATCATTCTCGCGAGGATAACCTGGACGACTTTATAGACGAAAAGCGCGGCGGTAAACCCTTCAGTTTCG
GCATCGGAAAACGAGGCTGGAAGCTAGCAACGGGCGAGACGGCCGTTTCCGGAAGAAGACCAAATGACGTTATCGGCCCGAAA
TATCTGCTCGGTTTGGGCAAAGGATTAGGCGAGGACGAAAATCTGAGTCAATAAAGCGTTTTCGTCGAGAAGCACGGATTGTT
CTCCTCC

>Lasius niger allatostatin precursor
MKSKTSLIAMRIIMFYLLSVVGRSTAAMEEAPLSSLHIPRLNPLSSNLEYDEPSEKRAYAYISEYKRLPLYNFGIGKRWIDNS
DDKRTRPFSFGIGKRLRDYRFGIGKRNSGYRPLGMDYFSVDNMEGYHSREDNLDDFIDEKRGGKPFSFGIGKRGWKLATGETA
VSGRRPNDVIGPKYLLGLGKGLGEDENLSQ*

>Lasius niger short neuropeptide F (sNPF) precursor
CCCCATCGAAGGCACAGCGGCTCCCCATATATATAAAGGGTCCCTCGGTATCGCCACACTTCGCATCATTACCGTCGTAACGT
GCGAGCCACTCAATCTCCGCTCGTCATCTGAGACTAAAGCTCAAGAATTCCAACGCGAGGATATGAGCACCATGTACGCTAAA
CGCTGCGCCGCTCTCGTTCTTCTCGTCGTGACAGTCGGCCTTATAAACGCCACCGAGAATTACATGGATTACGGAGAAGAGAT
GGCGGAGAAAACGCCCGCGGAGAACATCCACGAGTTCTACAGGCTTCTGCTGCAACGCAATGCGCTGGATAACGCAGGTTTCG
GTGGCATCCCGTTGGAGCACCTGATGATCCGCAAGTCGCAGCGATCGCCGTCGCTGCGTCTTCGATTCGGTCGTTCCGGACCG
CACGTCTCCGCGGGAGCTCTTCCAAGACCCGTGGGATACGAC

>Lasius niger short neuropeptide F (sNPF) precursor
MSTMYAKRCAALVLLVVTVGLINATENYMDYGEEMAEKTPAENIHEFYRLLLQRNALDNAGFGGIPLEHLMIRKSQRSPSLRL
RFGRSGPHVSAGALPRPVGYD…

Supplementary References

1. H. Hulme *et al.*, Basal ganglia neuropeptides show abnormal processing associated with L-DOPA-induced dyskinesia. *NPJ Parkinsons Dis* **8**, 41 (2022).

2. Y. L. Cintron-Diaz, M. E. Gomez-Hernandez, M. Verhaert, P. Verhaert, F. Fernandez-Lima, Spatially Resolved Neuropeptide Characterization from Neuropathological Formalin-Fixed, Paraffin-Embedded Tissue Sections by a Combination of Imaging MALDI FT-ICR Mass Spectrometry Histochemistry and Liquid Extraction Surface Analysis-Trapped Ion Mobility Spectrometry-Tandem Mass Spectrometry. *J Am Soc Mass Spectrom* **33**, 681-687 (2022).

3. K. DeLaney *et al.*, Mass Spectrometry Quantification, Localization, and Discovery of Feeding-Related Neuropeptides in Cancer borealis. *ACS Chem Neurosci* **12**, 782-798 (2021).

4. D. Cattani *et al.*, Perinatal exposure to a glyphosate-based herbicide causes dysregulation of dynorphins and an increase of neural precursor cells in the brain of adult male rats. *Toxicology* **461**, 152922 (2021).

5. D. C. Castro, Y. R. Xie, S. S. Rubakhin, E. V. Romanova, J. V. Sweedler, Image-guided MALDI mass spectrometry for high-throughput single-organelle characterization. *Nat Methods* **18**, 1233-1238 (2021).

6. E. A. Wood *et al.*, Neuropeptide Localization in Lymnaea stagnalis: From the Central Nervous System to Subcellular Compartments. *Front Mol Neurosci* **14**, 670303 (2021).

7. J. Habenstein *et al.*, Transcriptomic, peptidomic, and mass spectrometry imaging analysis of the brain in the ant Cataglyphis nodus. *J Neurochem* **158**, 391-412 (2021).

8. N. Q. Vu, A. R. Buchberger, J. Johnson, L. Li, Complementary neuropeptide detection in crustacean brain by mass spectrometry imaging using formalin and alternative aqueous tissue washes. *Anal Bioanal Chem* **413**, 2665-2673 (2021).

9. K. DeLaney, L. Li, Neuropeptidomic Profiling and Localization in the Crustacean Cardiac Ganglion Using Mass Spectrometry Imaging with Multiple Platforms. *J Am Soc Mass Spectrom* **31**, 2469-2478 (2020).

10. B. Chen, M. Vavrek, R. Gundersdorf, W. Zhong, M. T. Cancilla, Combining MALDI mass spectrometry imaging and droplet-base surface sampling analysis for tissue distribution, metabolite profiling, and relative quantification of cyclic peptide melanotan II. *Anal Chim Acta* **1125**, 279-287 (2020).

11. A. R. Buchberger, N. Q. Vu, J. Johnson, K. DeLaney, L. Li, A Simple and Effective Sample Preparation Strategy for MALDI-MS Imaging of Neuropeptide Changes in the Crustacean Brain Due to Hypoxia and Hypercapnia Stress. *J Am Soc Mass Spectrom* **31**, 1058-1065 (2020).

12. A. Ly *et al.*, Enhanced Coverage of Insect Neuropeptides in Tissue Sections by an Optimized Mass-Spectrometry-Imaging Protocol. *Anal Chem* **91**, 1980-1988 (2019).

13. S. Sheraz *et al.*, Enhanced Ion Yields Using High Energy Water Cluster Beams for Secondary Ion Mass Spectrometry Analysis and Imaging. *Anal Chem* **91**, 9058-9068 (2019).

14. M. Pratavieira *et al.*, MALDI Imaging Analysis of Neuropeptides in Africanized Honeybee (Apis mellifera) Brain: Effect of Aggressiveness. *J Proteome Res* **17**, 2358-2369 (2018).

15. Y. Zhang *et al.*, A Multifaceted Mass Spectrometric Method to Probe Feeding Related Neuropeptide Changes in Callinectes sapidus and Carcinus maenas. *J Am Soc Mass Spectrom* **29**, 948-960 (2018).

16. P. Sui *et al.*, Neuropeptide imaging in rat spinal cord with MALDI-TOF MS: Method development for the application in pain-related disease studies. *Eur J Mass Spectrom (Chichester)* **23**, 105-115 (2017).

17. E. Bivehed, R. Stromvall, J. Bergquist, G. Bakalkin, M. Andersson, Region-specific bioconversion of dynorphin neuropeptide detected by in situ histochemistry and MALDI imaging mass spectrometry. *Peptides* **87**, 20-27 (2017).

18. F. Schmitt, J. T. Vanselow, A. Schlosser, C. Wegener, W. Rossler, Neuropeptides in the desert ant Cataglyphis fortis: Mass spectrometric analysis, localization, and age-related changes. *J Comp Neurol* **525**, 901-918 (2017).

19. A. Hishimoto *et al.*, Molecular Histochemistry Identifies Peptidomic Organization and Reorganization Along Striatal Projection Units. *Biol Psychiatry* **79**, 415-420 (2016).

20. T. H. Ong *et al.*, Mass Spectrometry Imaging and Identification of Peptides Associated with Cephalic Ganglia Regeneration in Schmidtea mediterranea. *J Biol Chem* **291**, 8109-8120 (2016).

21. Y. Zhang, A. Buchberger, G. Muthuvel, L. Li, Expression and distribution of neuropeptides in the nervous system of the crab Carcinus maenas and their roles in environmental stress. *Proteomics* **15**, 3969-3979 (2015).

22. P. Sosnowski, T. Zera, B. Wilenska, E. Szczepanska-Sadowska, A. Misicka, Imaging and identification of endogenous peptides from rat pituitary embedded in egg yolk. *Rapid Commun Mass Spectrom* **29**, 327-335 (2015).

23. H. Ye *et al.*, Defining the Neuropeptidome of the Spiny Lobster Panulirus interruptus Brain Using a Multidimensional Mass Spectrometry-Based Platform. *J Proteome Res* **14**, 4776-4791 (2015).

24. S. Deb-Choudhury *et al.*, Direct localisation of molecules in tissue sections of growing antler tips using MALDI imaging. *Mol Cell Biochem* **409**, 225-241 (2015).

25. F. Schmitt *et al.*, Neuropeptidomics of the carpenter ant Camponotus floridanus. *J Proteome Res* 10.1021/pr5011636, Feb 2. [Epub ahead of print] (2015).

26. R. Chen, C. Ouyang, M. Xiao, L. Li, In situ identification and mapping of neuropeptides from the stomatogastric nervous system of Cancer borealis. *Rapid Commun Mass Spectrom* **28**, 2437-2444 (2014).

27. M. Pratavieira *et al.*, MALDI imaging analysis of neuropeptides in the Africanized honeybee (Apis mellifera) brain: effect of ontogeny. *J Proteome Res* **13**, 3054-3064 (2014).

28. C. Meriaux *et al.*, Human temporal lobe epilepsy analyses by tissue proteomics. *Hippocampus* **24**, 628-642 (2014).

29. Z. Zhang, S. Jiang, L. Li, Semi-automated liquid chromatography-mass spectrometric imaging platform for enhanced detection and improved data analysis of complex peptides. *J Chromatogr A* **1293**, 44-50 (2013).

30. R. M. Sturm, T. Greer, R. Chen, B. Hensen, L. Li, Comparison of NIMS and MALDI platforms for neuropeptide and lipid mass spectrometric imaging in C. borealis brain tissue. *Anal Methods* **5**, 1623-1628 (2013).

31. G. Thiery-Lavenant, A. I. Zavalin, R. M. Caprioli, Targeted multiplex imaging mass spectrometry in transmission geometry for subcellular spatial resolution. *J Am Soc Mass Spectrom* **24**, 609-614 (2013).

32. H. Ye, L. Hui, K. Kellersberger, L. Li, Mapping of neuropeptides in the crustacean stomatogastric nervous system by imaging mass spectrometry. *J Am Soc Mass Spectrom* **24**, 134-147 (2013).

33. J. P. Salisbury *et al.*, A rapid MALDI-TOF mass spectrometry workflow for Drosophila melanogaster differential neuropeptidomics. *Mol Brain* **6**, 60 (2013).

34. L. Mark, G. Maasz, Z. Pirger, High resolution spatial distribution of neuropeptides by MALDI imaging mass spectrometry in the terrestrial snail, Helix pomatia. *Acta Biol Hung* **63 Suppl 2**, 113-122 (2012).

35. P. Kallback, M. Shariatgorji, A. Nilsson, P. E. Andren, Novel mass spectrometry imaging software assisting labeled normalization and quantitation of drugs and neuropeptides directly in tissue sections. *J Proteomics* **75**, 4941-4951 (2012).

36. Z. Zhang, C. Jia, L. Li, Neuropeptide analysis with liquid chromatography-capillary electrophoresis-mass spectrometric imaging. *J Sep Sci* **35**, 1779-1784 (2012).

37. E. A. Jones *et al.*, Imaging mass spectrometry to visualize biomolecule distributions in mouse brain tissue following hemispheric cortical spreading depression. *J Proteomics* **75**, 5027-5035 (2012).

38. M. Zhong, C. Y. Lee, C. A. Croushore, J. V. Sweedler, Label-free quantitation of peptide release from neurons in a microfluidic device with mass spectrometry imaging. *Lab Chip* **12**, 2037-2045 (2012).

39. J. Hanrieder *et al.*, L-DOPA-induced dyskinesia is associated with regional increase of striatal dynorphin peptides as elucidated by imaging mass spectrometry. *Mol Cell Proteomics* **10**, M111 009308 (2011).

40. T. A. Zimmerman, S. S. Rubakhin, J. V. Sweedler, MALDI mass spectrometry imaging of neuronal cell cultures. *J Am Soc Mass Spectrom* **22**, 828-836 (2011).

41. P. Chansela *et al.*, Visualization of neuropeptides in paraffin-embedded tissue sections of the central nervous system in the decapod crustacean, Penaeus monodon, by imaging mass spectrometry. *Peptides* **34**, 10-18 (2012).

42. J. Bruand *et al.*, Automated querying and identification of novel peptides using MALDI mass spectrometric imaging. *J Proteome Res* **10**, 1915-1928 (2011).

43. A. Rompp *et al.*, Histology by mass spectrometry: label-free tissue characterization obtained from high-accuracy bioanalytical imaging. *Angew Chem Int Ed Engl* **49**, 3834-3838 (2010).

44. M. R. L. Paine, S. R. Ellis, D. Maloney, R. M. A. Heeren, P. Verhaert, Digestion-Free Analysis of Peptides from 30-year-old Formalin-Fixed, Paraffin-Embedded Tissue by Mass Spectrometry Imaging. *Anal Chem* **90**, 9272-9280 (2018).

45. R. Chen, L. Hui, S. S. Cape, J. Wang, L. Li, Comparative Neuropeptidomic Analysis of Food Intake via a Multi-faceted Mass Spectrometric Approach. *ACS Chem Neurosci* **1**, 204-214 (2010).

46. R. Chen *et al.*, Mass spectral analysis of neuropeptide expression and distribution in the nervous system of the lobster Homarus americanus. *J Proteome Res* **9**, 818-832 (2010).

47. T. A. Zimmerman, S. S. Rubakhin, E. V. Romanova, K. R. Tucker, J. V. Sweedler, MALDI mass spectrometric imaging using the stretched sample method to reveal neuropeptide distributions in aplysia nervous tissue. *Anal Chem* **81**, 9402-9409 (2009).

48. R. Chen, L. Hui, R. M. Sturm, L. Li, Three dimensional mapping of neuropeptides and lipids in crustacean brain by mass spectral imaging. *J Am Soc Mass Spectrom* **20**, 1068-1077 (2009).

49. E. B. Monroe *et al.*, SIMS and MALDI MS imaging of the spinal cord. *Proteomics* **8**, 3746-3754 (2008).

50. B. Li *et al.*, Genomics, transcriptomics, and peptidomics of neuropeptides and protein hormones in the red flour beetle Tribolium castaneum. *Genome Res* **18**, 113-122 (2008).

51. K. Jo *et al.*, Mass spectrometric imaging of peptide release from neuronal cells within microfluidic devices. *Lab Chip* **7**, 1454-1460 (2007).

52. S. S. DeKeyser, K. K. Kutz-Naber, J. J. Schmidt, G. A. Barrett-Wilt, L. Li, Imaging mass spectrometry of neuropeptides in decapod crustacean neuronal tissues. *J Proteome Res* **6**, 1782-1791 (2007).

53. A. F. Altelaar, J. van Minnen, C. R. Jimenez, R. M. Heeren, S. R. Piersma, Direct molecular imaging of Lymnaea stagnalis nervous tissue at subcellular spatial resolution by mass spectrometry. *Anal Chem* **77**, 735-741 (2005).

54. S. Neupert, R. Predel, Mass spectrometric analysis of single identified neurons of an insect. *Biochem Biophys Res Commun* **327**, 640-645 (2005).

55. P. Verleyen *et al.*, Neuropeptidomics of the grey flesh fly, Neobellieria bullata. *Biochem Biophys Res Commun* **316**, 763-770 (2004).

56. S. S. Rubakhin, W. T. Greenough, J. V. Sweedler, Spatial profiling with MALDI MS: distribution of neuropeptides within single neurons. *Anal Chem* **75**, 5374-5380 (2003).

57. J. Habenstein, M. Thamm, W. Rossler, Neuropeptides as potential modulators of behavioral transitions in the ant Cataglyphis nodus. *J Comp Neurol* **529**, 3155-3170 (2021).

58. J. Gospocic *et al.*, The Neuropeptide Corazonin Controls Social Behavior and Caste Identity in Ants. *Cell* **170**, 748-759 e712 (2017).

59. Z. Liutkeviciute *et al.*, Oxytocin-like signaling in ants influences metabolic gene expression and locomotor activity. *FASEB J* **32**, fj201800443 (2018).

60. G. Baggerman, A. Cerstiaens, A. De Loof, L. Schoofs, Peptidomics of the larval Drosophila melanogaster central nervous system. *J Biol Chem* **277**, 40368-40374 (2002).

61. M. Sterkel, H. Urlaub, R. Rivera-Pomar, S. Ons, Functional proteomics of neuropeptidome dynamics during the feeding process of Rhodnius prolixus. *J Proteome Res* **10**, 3363-3371 (2011).

62. P. Bajracharya, H. L. Lu, P. V. Pietrantonio, The red imported fire ant (Solenopsis invicta Buren) kept Y not F: predicted sNPY endogenous ligands deorphanize the short NPF (sNPF) receptor. *PLoS One* **9**, e109590 (2014).

63. L. Bestea *et al.*, The short neuropeptide F regulates appetitive but not aversive responsiveness in a social insect. *iScience* **25**, 103619 (2022).

64. B. Han *et al.*, Tachykinin signaling inhibits task-specific behavioral responsiveness in honeybee workers. *Elife* **10** (2021).

65. A. Brockmann *et al.*, Quantitative peptidomics reveal brain peptide signatures of behavior. *Proc Natl Acad Sci U S A* **106**, 2383-2388 (2009).

66. A. Kaiser *et al.*, A three-dimensional atlas of the honeybee central complex, associated neuropils and peptidergic layers of the central body. *J Comp Neurol* **530**, 2416-2438 (2022).
